# Highly-sensitive quantification of carbamazepine and identification of its degradation and metabolism products in human liver by high performance liquid chromatography – High resolution mass spectrometry

**DOI:** 10.1016/j.toxrep.2025.101923

**Published:** 2025-01-27

**Authors:** Andrei Pirogov, Ekaterina Shirokova, Samvel Barsegyan, Nikita Gandlevskiy, Valeriya Akimova, Alessandro Barge, Aleksander Nosyrev

**Affiliations:** a Department of chemistry, M.V. Lomonosov Moscow State University, Chair of Analytical Chemistry, Leninskiye gory str. 1/3, Moscow 119991, Russia; b Federal State Budgetary Institution «Russian Centre of Forensic Medical Expertise» of the Ministry of Health of the Russian Federation, Polikarpov str, 12/13, Moscow 125284, Russia; c Department of Drug Science and Technology, Turin University, via P. Giuria 9, Turin 10125, Italy; d FSAEI of Higher Education I.M. Sechenov First Moscow State Medical University (Sechenov University), Trubetskaya str, 8/2, Moscow 119048, Russia

**Keywords:** Carbamazepine, Liver metabolism, HPLC-HRMS

## Abstract

A method for the qualitative and quantitative determination of carbamazepine in human *post mortem* liver tissues using high-performance liquid chromatography coupled with high-resolution mass spectrometry has been developed. Validation has been carried out and the main analytical characteristics of the developed method have been determined. The limit of detection (LOD) is 1 ng/g, and the lower limit of quantification (LLOQ) is 5 ng/g. The range of working concentrations for the calibration curve is 5–2000 ng/g. When assessing analyte carryover, the analyte signal of the sample does not exceed 20 % of the signal at the LLOQ level. Degradation products of carbamazepine in model solutions were studied under the presence of hydrochloric acid, sodium hydroxide, and hydrogen peroxide oxidation. Twenty-two degradation products were identified. It was found that the most intensive degradation process of carbamazepine, resulting in various degradation products, is observed during its oxidation with an acidified solution of 3 % hydrogen peroxide at pH= 1–2. The stability of carbamazepine in liver tissues was studied during storage under ambient conditions over various periods. The maximum concentration decline is observed during the first week of storage (on average by 20 %), and then the concentration approximately halves over 8 weeks. Based on the analysis of forensic samples from human liver, 2 out of the 22 carbamazepine degradation products described in this study were detected.

## Highlights

• A novel HPLC-HRMS method quantified carbamazepine in human cadaveric liver samples.
• The artificial aging of carbamazepine was performed giving 22 degradation products.
• The stability of carbamazepine in liver tissues was assessed.
• 2 out of the 22 identified degradation products detected in human liver tissues.

## Graphical Abstract

**Figure:**
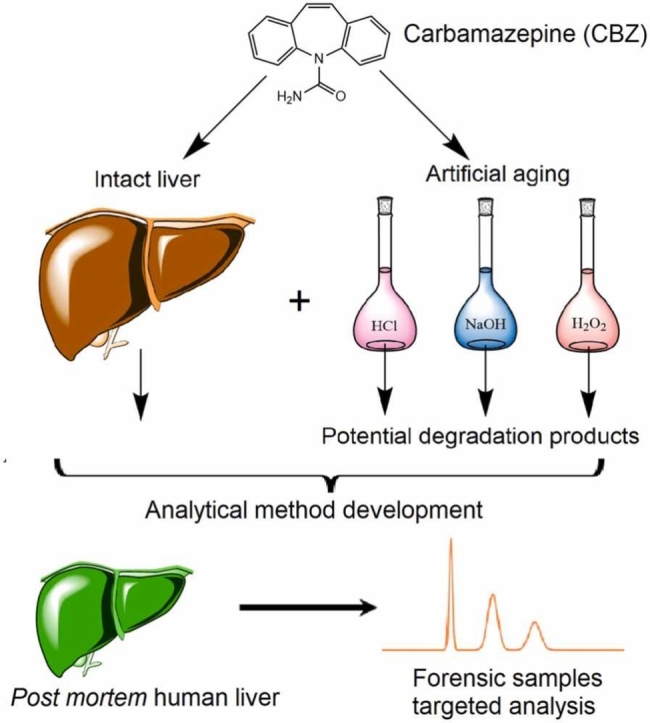

## 1 Introduction

**Fig. 1:**
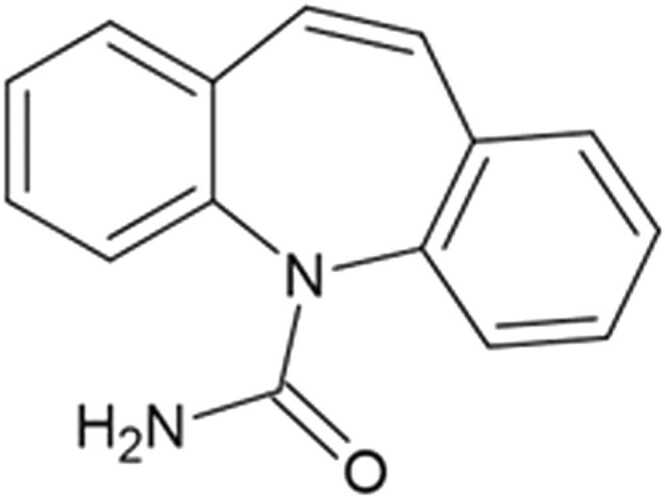
Structural formula of carbamazepine.

Carbamazepine (CBZ), or 5H-dibenzo[*b*,*f*]azepine-5-carboxamide, is an antiepileptic and anticonvulsant drug frequently used for neurological and psychiatric patients to treat seizures, neuropathic pain, or bipolar disorder (Fig. 1). CBZ, in its chemical structure, is an iminostilbene derivative, contains a carbamyl group, and is structurally similar to tricyclic antidepressants. This drug is also of interest to forensic medicine and chemical-toxicological analysis specialists, as uncontrolled use and overdose of carbamazepine can result in fatal outcomes. Due to the close proximity of the toxic dose to the therapeutic values for carbamazepine, cases of both intentional and accidental poisonings are known. According to the N.V. Sklifosovsky Research Institute of Emergency Medicine, there has been a significant increase in the number of fatal carbamazepine poisonings, including among children, over the past five years.

Additionally, one of the reasons for the forensic toxicology interest in antiepileptic drugs is the significant number of sudden unexplained deaths among people with epilepsy who take these medications [1]*.* In conducting forensic investigations of deaths caused by poisoning, it is necessary to accurately identify and quantify the intoxicant and the dose received. For this purpose, a reliable detection method and a validated quantitative determination methodology for pharmaceutical drugs, their metabolites, and degradation products in biological material are required. The challenge in implementing such methods lies in the high degradation rate of the drug, both under adverse environmental conditions (during storage) and during sample preparation and analysis.

The aim of this work is to develop and validate a highly sensitive technique for the quantification of carbamazepine, as well as the identification of its metabolites and degradation products in human biological material, using high-resolution chromatographic-mass spectrometry. This methodology is intended to address expert tasks in forensic toxicology.

After administration, CBZ binds to plasma proteins at a rate of 75–85 %, and its elimination is almost entirely dependent on biotransformation in the liver through epoxidation and hydroxylation. It is assumed that carbamazepine-epoxide (CBZ-EP), being an active metabolite, contributes to the observed therapeutic and side effects. The study [2] also showed that CBZ is almost completely metabolized in the liver, with only about 5 % of the drug excreted unchanged. The main metabolic pathways have been identified [3].

Therefore, when working with biological material containing CBZ and collected *post mortem*, it is necessary to consider the time elapsed since the presumed intake of CBZ and its elimination pathways. Thus, the liver was chosen as the matrix carrier for this work and for the development of a quantitative determination method for CBZ.

The literature presents numerous methods aimed at the quantitative determination of CBZ in various matrices (Table 1). However, due to the specific requirements of chemical-toxicological forensic examinations, only some of these methods are suitable for forensic purposes.

**Table 1:** Conditions for the quantification of carbamazepine in various samples using LC.

Sample	Sample preparation	Separation and detection conditions	Analytical characteristics	Source
Hair	Liquid-liquid extraction Extraction by the mixture MeOH/ MeCN/ 2 мМ of ammonium formate in 8 % MeCN, pH= 5,3 (25:25:50 v/v/v). 18 hours of incubation.	Column: ACQUITY UPLC HSS C18 (150 mm × 2.1 mm, 1.8 μm) Т = 50°C Flow rate: 0.4 mL/min MP А: 5 мМ ammonium formate (pH 3.0) MP В: 0.1 % formic acid in MeCN MS/MS, triple quadrupole (MRM mode, ESI)	LLOQ < 5.0 pg/mg Accuracy 75–125 %	[4]
Blood serum	Blood samples were centrifugated, 1 mL of serum has been sampled. 30 μL of sample were mixed with 150 μL of IS, then mixed and centrifuged. 40 μl of the supernatant was placed into a vial with 160 μL of 0.1 % formic acid.	Column: Phenomenex Torrance Luna C18 (50 × 2,0 mm, 5 μm, 100 Å) Т = 25°C Flow rate: 0,4 mL/min MP А: 0.1 % of formic acid MP В: MeCN MS/MS, triple quadrupole (ESI+)	LOD = 0.8 ng/mL LLOQ = 2.4 ng/mL Recovery: 96–98 %	[5]
Blood serum	Protein precipitation was carried out in a 96-well plate. To 100 μl of plasma, 20 μl of IS (phenacetin, 100 ng/mL) and 300 μl of MeCN were added. The samples were mixed, placed in a positive pressure device (413 kPa, 5 min), the supernatant was transferred to other tubes and evaporated in a stream of nitrogen at 45°C. Redissolved in 100 µl of MP.	Column: Agilent Eclipse XDB-C18 (2.1 × 50 mm, 1.8 μm) Т = 25°C MP А: MeCN MP В: 0,1 % formic acid Gradient: 15 %A→30 %A from 0 to 3 min 30 %A→95 %A from 3 to 5 min MS/MS, triple quadrupole (ESI+)	LLOQ = 50.0 ng/mL Recovery 74,7–93,5 %	[6]
Blood plasma	Liquid-liquid extraction To 500 μL of plasma, 500 μL of 0.1 M NaOH and IS (nitrazepam 800 ng/mL in H_2_O) were added. Then, 3 mL of ethyl acetate was added to the mixture, shaken and centrifuged at 10°C. The aqueous phase was collected and frozen at −30°C for 3 min. The organic phase was evaporated under a nitrogen stream at 40°C. The dry residue was redissolved in the mobile phase.	Column: Phenomenex Luna C18 (150 × 2 mm, 5 μm) Т = 25°C Flow rate: 0.25 mL/min MP: MeCN/ MeOH/ 0.1 % formic acid (10:70:20 v/v/v) MS/MS, triple quadrupole (ESI+)	LLOQ = 0.72 ng/mL t_R_: 2.65–2.84 min	[7]
Blood plasma	Liquid-liquid extraction Standard solutions were dissolved in MeCN. 300 μL of acetone was added to plasma samples (protein denaturation). Centrifuged and evaporated at 37°C. The dry residue was dissolved in a mixture of MeCN:water (50:50 v/v)	Column: Zorbax eclipse XD8 C8 (150 × 4.6 mm, 4 μm) Т = 25°C Flow rate: 0.8 mL/min MP А: MeCN MP В: formate buffer (2 mM, pH 3) consisted of formic acid salt (126 mg/L) in purified water. MS/MS, triple quadrupole (ESI+)	LLOQ = 500 ng/mL Accuracy 100 ± 20 % Recovery 90 ± 6.7 %	[8]
Shellfish	Liquid-liquid extraction 10 mL of MeCN and 5 mL of H_2_O, followed by the pack of acetate salts were added to the tissue sample. Centrifuged. The supernatant was evaporated to dryness at 40 °C under N_2_ flow. The dry residue was dissolved in 50 μL of^13^C-phenacetin and diluted to 500 μL with water	Column: Poroshell 120 SB C8 (50 × 2.1 mm, 2.7 μm) Т = 60°C Flow rate: 0.6 mL/min MP А: 0.01 % acetic acid in water MP В: MeCN MS/MS (MRM mode, ESI(+).	LOD = 0.04 ng/g LLOQ < 5.0 ng/g Accuracy 100 ± 20 %	[9]
Brain tissue	Liquid-liquid extraction 1 mL of IS solution was added to tissue samples (20–200 μg), centrifuged, and homogenized. Extracted with buffer solution (pH=6.0): 20.0 g sodium dihydrogen phosphate 2-hydrate, 4.5 g di-sodium hydrogen phosphate 2-hydrate, and 1.5 g sodium azide in 1 l of deionized water. 1 mL of acetone was added to 2 mL of sample, shaken, and centrifuged. 1 mL of extraction buffer was added to the supernatant. Afterwards, extraction was carried out with a mixture of 970 mL of dichloromethane with 30 mL of 2-propanol.	Column: С18 ODS (200 × 2.1 mm, 5 μm) Т = 65°C Flow rate: 0,3 mL/min MP А: 0.05 M NH_4_H_2_PO_4_ buffer (pH=4.4) / MeCN (90:10 v/v) MP В: 0.05 M NH_4_H_2_PO_4_ buffer (pH=4.4) / MeCN (40:60) UV detection (207 nm)	LOD = 870 ng/mL	[10]
Fish liver	Liquid-liquid extraction 10 mL of hexane was added to 2 g of liver sample, mixed, and centrifuged. The extraction was repeated 3 times, and the extracts were combined. Evaporated under a stream of nitrogen. The dry residue was dissolved in 2 mL of 90 % MeCN, diluted with 50 mL of water and purified using solid phase extraction through a cartridge (Oasis HLB) by washing with 5 mL of methanol and 5 mL of water. The methanol fraction was sifted out and the residue was dissolved in 4 mL of 90 % MeCN	Column: Alltima С18 (250 × 4.6 mm, 5 μm) Т = 25°C Flow rate: 1 mL/min MP А: Water MP В: MeCN MS/MS (MRM mode, ESI(-).	LLOQ = 4.2 ng/g Recovery 75–85 %	[11]
Human liver (metabolomic study)	Liquid-liquid extraction Liver samples of 10 g were placed in 10 mM potassium phosphate buffer (pH = 7.4) containing 0.9 % NaCl. Then, the tissue was homogenized to obtain microsomes in a buffer supplemented with 0.25 M sucrose. Centrifuged. The supernatant containing microsomal protein was carefully separated from the glycogen granules, resuspended in a mixture of 10 mM KP buffer (pH = 7.4): 1 mM EDTA: 1.15 % KCI and centrifuged again. The washed pellet was resuspended in either 50 mM KP buffer (pH=7.4) or 100 mM Tris HCI buffer (pH=7.4). IS - 2-methyl-CBZ (11.3 µg/mL)	Column: Zorbax C8 (4.6 × 250 mm, 5 μm) Т = 50°C Flow rate: 1.5 mL/min MP А: MeOH MP В: H_2_O UV detection (210 nm)	LLOQ = 50 µM	[12]
Fish tissues	QuEChERS IS and 10 mL of MeCN were added to 1 g of homogenized tissue. Shaked for 10 min. A mixture of salts from the QuEChERS kit (4 g MgSO_4_/1 g NaCl) was added, shaken for 1.5 min, and centrifuged (5 min, 4000 rpm). 2 mL of organic layer was collected and transferred to a test tube containing 50 mg of Z-Sep+ Bulk salt. Mixed and centrifuged. The supernatant layer was collected and evaporated to dryness (40°C). Redissolved in 500 μL of 0.1 % formic acid solution in a water/methanol mixture (10:90 v/v)	Column: Hypersil Gold C18 (2.1 × 100 mm, 1.9 μm) Т = 35^о^С Flow rate: 250 μL/min MP А: 0.1 % formic acid in water MP В: 0.1 % formic acid in MeOH MS/MS, Orbitrap XL Fourier (full scan mode, ESI+).	LLOQ = 2.5 ng/g Recovery 97 %	[13]

IS – internal standard, MP – mobile phase, LLOQ – low limit of quantification, LOD – limit of detection, t_R_ – retention time.

Using HPLC-MS allows for the avoidance of degradation that might occur with GC-MS. HPLC-MS provides more accurate results and can detect lower concentrations of substances in samples. GC has several limitations, including the need for analytes to be volatile and non-polar. Under normal GC operating conditions, CBZ undergoes thermal degradation in the injector, forming iminostilbene (IM) and 9-methylacridine, which sometimes necessitates an additional derivatization step for accurate analysis [14], [15]. Furthermore, GC is highly sensitive to biological contaminants such as saturated lipids, sterols, and fatty acids, which negatively impact the lifespan of consumables.

Analysing the data presented in the table, it was found that the primary sample preparation method for the subsequent determination of CBZ in tissues is liquid-liquid extraction (LLE). Based on the physicochemical properties of CBZ and the literature, the optimal solvents for the extraction of CBZ from biological tissues are acetonitrile and ethyl acetate [13]. In modern laboratory practice, solid-phase extraction using the QuEChERS method is increasingly employed [16], [17], [18].

Methodologies utilizing HPLC-MS/MS demonstrate superior LODs and LOQs of CBZ. According to the literature, the LOD using a mass spectrometric detector is generally an order of magnitude lower. The influence of the biological matrix is minimized, making this method the most relevant for forensic toxicology work.

The relevance of our work is further supported by the fact that there are comparatively few scientific studies available to us that propose a methodology for the qualitative and quantitative determination of CBZ in biological material (liver) for forensic purposes using the HPLC-MS/MS method.

## 2 Materials and methods

### 2.1 Reagents

The following reagents were used during the study and sample preparation: acetonitrile (Fisher Chemicals Optima LC/MS grade), formic acid (98–100 %, Merck, Germany), ammonium formate (for LC-MS, Fluka analytical, Germany), hydrochloric acid HCl (extra pure, Khimmed, Russia, GOST 3118–77rev1), hydrogen peroxide (37 %, med. Khimmed, Russia, GOST 177–88rev1), sodium hydroxide NaOH (extra pure, Khimmed, Russia, GOST 4328–77rev.1,2), ethyl acetate (for LC-MS, J.T.Baker, USA).

### 2.2 Equipment and methods

Chromatographic separation was performed on a Vanquish-VH-C10A HPLC system (ThermoScientific, USA) using a Kinetex C18 column (50 mm × 3 mm, 2.6 μm, 100 Å, Phenomenex, USA) with an Acquity UPLC BEH C18 1.7 μm precolumn (Waters, USA). The chromatographic conditions are provided in Table 2. Detection of CBZ and its degradation products in biological samples was conducted using an Orbitrap Exploris 120 mass spectrometer (ThermoScientific™, USA) with heated electrospray ionization (H-ESI). Chromatograms were acquired in Full Scan mode with ion fragmentation in the mass range of 100–1000 Da. The ionization source parameters are were set as follows: vaporizer temperature – 400 °C, ion transfer tube temperature – 320 °C, transition dwell time – 0.05 sec, spray voltage – positive ion 3500 V, negative ion – 2500 V, Intensity threshold – 1.0e4, HCD collision energies – 15, 30, 45 eV. Metabolite identification was performed using Compound Discoverer™ software, Thermo FreeStyle™ (Thermo Scientific™, USA), as well as the software packages Competitive Fragmentation Modeling (CMF-ID) and MetFrag (Thermo Scientific™). Identification of CBZ degradation products relied on parameters such as accurate mass of the hydrated molecular ion (to ten-thousandths of a dalton), isotopic distribution (MS), and characteristic ion fragmentation (MS2).

**Table 2:** Chromatographic conditions.

Parameter	Values
MP А	0.1 % formic acid, 2 мМ ammonium formate, 1 % MeCN in H_2_O
MP B	0.1 % formic acid, 2 мМ ammonium formate, 1 % H_2_O in MeCN
Column temperature	40ºС
Injection volume	5 μL
Flow rate	0,4 mL/min
Run time	15 min
Gradient programme	time, minMP А, % 0.098 1.098 10.02 12.02 12.198 15.098

## 3 Materials and equipment used in sample preparation

Extraction was carried out using the VetexQ-tox IL-5550–4956 extraction kit, 15 mL Pyrex glass tubes with screw caps (Corning, UK), 1.5 mL Eppendorf tubes (Eppendorf, Germany), mechanical pipettes (Eppendorf research plus, Eppendorf, Germany), analytical balances (Mettler Toledo XS204, USA), Vortex microcentrifuge (ELMI V-3, Latvia), orbital shaker (BioSan PSU-10i, Latvia), centrifuge (ELMI CM-6M, Latvia), centrifuge (Eppendorf AG 22331, Germany), and water purification system (Millipore Direct-Q 5 UV, USA).

### 3.1 Accelerated aging of carbamazepine

Methanol solutions containing 1 mg of CBZ were placed in nine Eppendorf-type tubes and evaporated in a concentrator for 15 minutes. The tubes were divided into sets of three, and 3 mL of 18 % hydrochloric acid HCl, 2 M sodium hydroxide NaOH solution, and 3 % H_2_O_2_ solution were added to each set, respectively. All samples were heated for various durations (10, 30, 60 minutes) in a boiling water bath, followed by acidification to pH= 1–2 with 0.1 M HCl. Extraction with 2 mL of ethyl acetate was performed, and the organic layer was collected. The aqueous phase was basified to pH= 10 with sodium hydroxide solution. Then, 2 mL of ethyl acetate was extracted again, and the supernatant was collected. Acidic and alkaline extracts were combined and evaporated. The dry residues were reconstituted in 150 μL of a mixture of 10 % acetonitrile - 0.1 % formic acid and transferred to 2 mL glass vials (Table 3).

**Table 3:** Sample preparation for performing of artificial aging.

Samples № 1–3			№ 4–6				№ 7–9		
1	2	3	4	5		6	7	8	9
+ 3 mL 18 % HCl			+ 3 mL 2М NaOH				+ 3 mL 3 % solution of H_2_O_2_, acidified by HCl to pH= 1–2		
Heating in water bath (100^о^С)									
10 min	30 min	60 min	10 min	30 min		60 min	10 min	30 min	60 min
Acidification by 0.1 М HCl to pH= 1–2									
Extraction by 2 mL of ethyl acetate									
Aqueous phase					Organic phase				
+ 2М NaOH to рН = 10									
Extraction by 2 mL of ethyl acetate									
Unification of organic extracts									
Evaporation 30 min									

### 3.2 Liver tissue sampling

During this study, forensic samples of biological material provided by the Federal State Budgetary Institution "RCMSE" of the Ministry of Health of Russia were used. Samples not containing CBZ were included for validation of the quantitative determination method, as well as two cadaveric liver samples obtained during pathological autopsies. To preserve the confidentiality of the investigation and medical secrecy, these samples will be referred to as FSE_1 and FSE_2 in the future. Blank liver samples were collected to validate the methodology for quantitative determination, with a standard CBZ solution of the corresponding concentration added to them. Two biological liver samples from cadavers FSE_1 and FSE_2 were used for the following purposes:

1. Conducting an experiment to investigate the stability of CBZ under environmental conditions;
2. Qualitative and quantitative determination of CBZ;
3. Qualitative analysis of CBZ degradation and metabolism products, as well as extraneous compounds.

### 3.3 Preparation of liver tissue samples

A 4.0 g portion of intact liver was homogenized, transferred to an extraction tube, and 200 μL standard working solution of CBZ (1 mg/mL) and 10 μL of internal standard (amitriptyline, 1 mg/mL) were added. The mixture was shaken and left for 15 minutes at room temperature. This compound has structural similarity to CBZ and similar physicochemical properties (M = 277.4 g/mol, LogP = 5, pKa = 9.5), thus comparable retention time on the column under the chosen chromatographic separation conditions (t_r_ = 5.6 min). A mixture of salts (1000 mg MgSO_4_ and 250 mg NaCl) was added, followed by 5 mL of acetonitrile, and vigorously shaken. The mixture was then subjected to ultrasonic bath treatment for 25–30 minutes and centrifuged for 5 minutes at 3000 rpm. The supernatant (3–3.5 mL) was transferred to a cleanup tube containing MgSO_4_ and sorbent. After shaking for 15 minutes and centrifuging for 5 minutes at 3000 rpm, 100 μL of the supernatant was transferred to a chromatographic vial and diluted with 900 μL of water.

### 3.4 Stability assessment

To assess the stability of CBZ in liver tissues containing CBZ, samples were kept at room temperature (25°C) with oxygen and light exposure for 1–8 weeks. A portion of the samples was collected weekly for analysis. After the specified time, the samples were frozen at −20°C for further storage. After 8 weeks, all frozen samples were thawed, and sample preparation was performed according to the general scheme, except for the addition of CBZ standard. In total, 20 samples were analysed in this manner.

## 4 Results and discussion

Within the framework of developing a technique for qualitative and quantitative analysis of carbamazepine by LC-MS/MS in human liver, the method of sample preparation, internal standard selection, and analysis conditions were determined.

A reverse-phase chromatography approach with a C18 column and mobile phases based on water and acetonitrile was chosen. High-resolution mass spectrometry was essential for retrospective analysis, allowing assessment of the drug's transformation processes over time. Determining the accurate masses of molecular ions enabled hypotheses regarding the compound's molecular formula, while MS/MS fragmentation spectra provided insights into the molecule's structure. An orbital trap-based mass spectrometric detector was selected to achieve the analysis objectives.

**Validation and specificity** were ensured through method validation referencing regulatory documents governing validation procedures in forensic chemical and chemico-toxicological analysis of biological material [19], [20]. Parallel measurements were conducted for five analytical cycles, each repeated twice. Following analysis of the calibration sample with the highest concentration per cycle, an intact liver sample (Blank) was introduced to control for sample carryover.

**Fig. 2:**
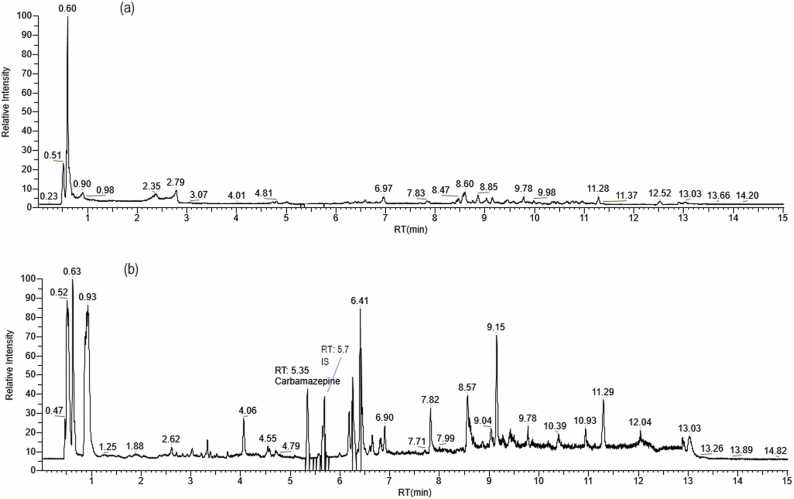
Comparison of chromatograms of liver extracts with addition of CBZ and AMT (a) and intact (b).

Specificity was evaluated by comparing chromatograms obtained from intact liver samples (Blank) with those from samples spiked with standard solutions of CBZ and internal standards. Chromatograms and mass spectra of intact liver samples showed absence of peaks corresponding to CBZ and AMT retention times (Fig. 2).

To construct the calibration model, calibration solutions were prepared using pure liver samples spiked with CBZ and an internal standard to the required concentrations. Calibration levels were set at 5, 10, 20, 50, 100, 500, 750, 1000, and 2000 ng/g. Control levels were set at 15, 800, and 1600 ng/g (QCL, QCM, QCH levels). The concentration of the internal standard was kept constant at 400 ng/g. The concentration level corresponding to the LLOQ of the method should allow for concentration determination ≤ 5 % of the maximum concentration. Control levels were defined as follows: lower level (L) – three times the LLOQ, middle level (M) – the average between L and H, approximately 30–50 % of the upper limit of the determined concentrations, upper level (H) – 80 % of the upper limit of the determined concentrations. The calibration curve, the equation and correlation coefficient are presented in Fig. S4.

The obtained calibration points were well described by a quadratic relationship. A linear relationship was observed in the low concentration range (5–100 ng/g); however, for our forensic medical practice needs, we expanded the analytical range to cover the maximum therapeutic concentrations of the drug in tissues. Due to the complexity of working with biological material and its limited accessibility in some cases, and because CBZ concentrations in individual expert samples can reach toxic or lethal levels exceeding 120.000 ng/g, it is not always feasible to use dilution methods to work within the linear range. It should be noted that presenting the curve as two linear segments is impractical due to vastly different matrix effects at high and low CBZ concentrations. Thus, the analytical range of the developed method is 5–2000 ng/g. Concentration deviations of calibration samples from nominal values did not exceed acceptable limits ( ± 20 %) across the entire calibration range. The LOD for CBZ was experimentally determined to be 1 ng/g (Table S4). Relative standard deviation and accuracy are presented in Table 4.

**Table 4:** Calculated parameters of the accuracy and precision of the method within the fifth analytical cycle. SD – sample standard deviation, RSD – relative standard deviation (%), E – accuracy (%), (n = 2, P = 0.95).

Nominal concentration, ng/g	Measured concentration, ng/g Average ± SD	RSD, %	Е, %
5	5.1 ± 0,3	6.15	2.37
10	10.9 ± 0,4	3.35	8.58
20	19.8 ± 0,3	1.75	−1.17
50	47 ± 6	13.38	−6.69
100	104 ± 13	13.05	4.55
500	524 ± 37	6.99	4.82
750	760 ± 75	9.89	1.17
1000	1040 ± 84	8.10	4.19
2000	2004 ± 138	6.92	0.19

### 4.1 Accuracy and precision

The determination of method accuracy and precision was conducted by analysing quality control samples at different levels: L, M, H with concentrations of 15.0, 800.0, and 1600.0 ng/g respectively. Accuracy was assessed using the relative error (E, %) as follows:

$$E\;\left(\%\right)=\left(\frac{\overset{®}{C}-C_{k\mathrm{nown}}}{{С}_{k\mathrm{nown}}}\right)*100\%,$$

where $\overset{®}{С}$ is the average concentration value within or between analytical cycles.

Precision (convergence) was evaluated using the relative standard deviation (RSD, %):

$$\mathrm{RSD}\left(\%\right)=\frac{\mathrm{SD}}{\overset{®}{C}}*100\%$$

where SD is the standard deviation of the analytical signal.

Both parameters must meet the requirements for analytical methods used in forensic chemical and chemico-toxicological analysis of biological materials, not exceeding 20 % for all concentration levels L, M, H. Table 5 presents the calculation of accuracy and precision for the quality control samples within the first analytical cycle. Precision parameters calculated for series of parallel measurements using the one-factor dispersion analysis method are presented in Table 6.

**Table 5:** Calculated accuracy and precision parameters for quality control samples within the first analytical cycle (n = 3, P = 0.95).

Sample	Nominal concentration, ng/g	Measured concentration, ng/g	Average, ng/g	SD	RSD, %	E, %
QC_L_1	15	15	16	1.7	10.6	9.2
QC_L_2		18				
QC_L_3		16				
QC_M_1	800	962	804	138.8	17.3	0.5
QC_M_2		748				
QC_M_3		702				
QC_H_1	1600	1774	1744	42.7	2.5	9.0
QC_H_2		1695				
QC_H_3		1764

**Table 6:** Accuracy and precision for the developed procedure in five analytical runs. AC – acceptance criterion.

Concentration level, ng/g	15	800	1600	AC
Convergence within a series of parallel quantifications (RSD, %)	16.8	16.6	4.1	±20.0 %
Convergence between series of parallel quantifications (RSD, %)	15.5	17.8	3.8	
Accuracy between series of parallel quantifications (Е, %)	8.7	18.5	7.1	

### 4.2 Sample transfer effect

According to [21], during method development, it is crucial to consider and minimize the transfer of the analyte from sample to sample. During validation, the transfer effect was assessed by introducing blank samples after samples with high concentrations or upper-level calibration solutions. To evaluate sample transfer during sequential sample analysis after the maximum concentration calibration sample (2000 ng/g, calibration level 9), and after the quality control sample H with a concentration of 1600 ng/g, an analysis of a CBZ-free sample (Blank) was conducted. Analysis of peak area ratios showed minimal substance transfer, meeting acceptability criteria (AC): the analyte signal of the sample did not exceed 20 % of the signal at the LLOQ level, and the IS signal of the sample did not exceed 5 % of the IS signal.

### 4.3 Stability study of CBZ under environmental storage conditions

Stability studies are conducted to model situations that may occur in natural and laboratory environments, which can affect the properties of the analyte to some extent [19]. It is known that CBZ is highly susceptible to thermal and chemical degradation, as well as photodegradation, raising concerns about its stability. When working with biological material collected for forensic medical analysis, understanding the stability of CBZ in tissue during storage under environmental conditions over different periods of time is crucial.

**Fig. 3:**
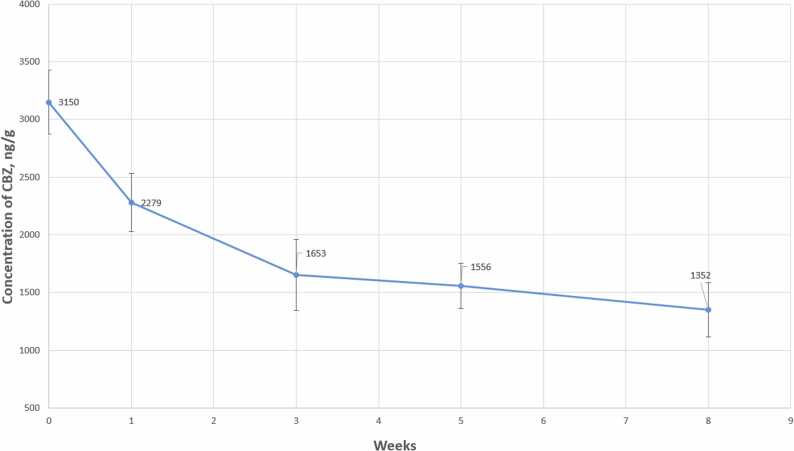
Graph of changes in CBZ concentration in liver samples versus storage time.

During an 8-week experiment, forensic liver samples (FSE_1, FSE_2) were kept under room conditions (25°C, oxygen access, light). Upon analysis of liver sample FSE_1, the initial CBZ concentration was found to be 3150 ± 280 ng/g. This concentration exceeds the therapeutic range for CBZ concentrations in liver tissue, indicating a potential lethal poisoning. Two samples were analyzed with two replicates each, and the average result, error, and relative standard deviation (RSD) were assessed. The analysis results are presented in Table S1 (Appendix). The data obtained were plotted graphically (Fig. 3).

Upon analysis of liver sample FSE_2, the initial CBZ concentration was found to be 7880 ± 820 ng/g. Both analyzed forensic liver samples initially contained CBZ amounts corresponding to toxic concentrations for this compound in tissue. For samples FSE_1 and FSE_2, the upper limit of the therapeutic concentration range was exceeded by 1.5 and 4 times, respectively. It can be hypothesized that individual or combined poisoning by the investigated substance could have led to the onset of death.

### 4.4 Study of degradation products after artificial aging

Accelerated aging tests are conducted in artificial aggressive environments and under simulated unfavourable storage conditions of drugs to establish their degradation products. Data from artificial aging simplify the process of identifying degradation products in post-mortem samples and enable forensic experts to draw conclusions about the ante-mortem use of the medicinal product and its approximate dosage. The information on degradation pathways plays a crucial role in cases involving the discovery of bodies in decomposition stages or in the analysis of exhumed biomaterials.

Table S3 in the Supplementary presents the identified degradation products of CBZ, their monoisotopic masses [M], accurate masses of protonated [M+H]^+^ and deprotonated [M-H]^-^ ions, structural formulas, and mass spectra. In our search for degradants, we relied on literature data, information from the "HMDB" database [22], and results from previous work using GC-MS methods [18].

Upon analysing the obtained data, it was found that the identified degradation products were mostly characteristic of all pathways of artificial aging. However, the concentration of each product varied significantly in different environments. An exception was iminodibenzyl, which was present in all conditions except those with the oxidizing agent H_2_O_2_.

**Fig. 4:**
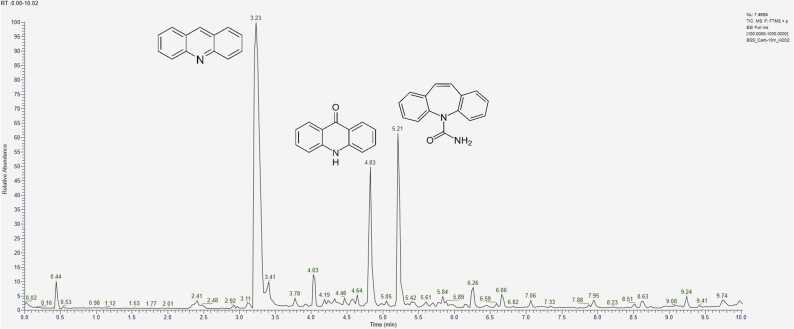

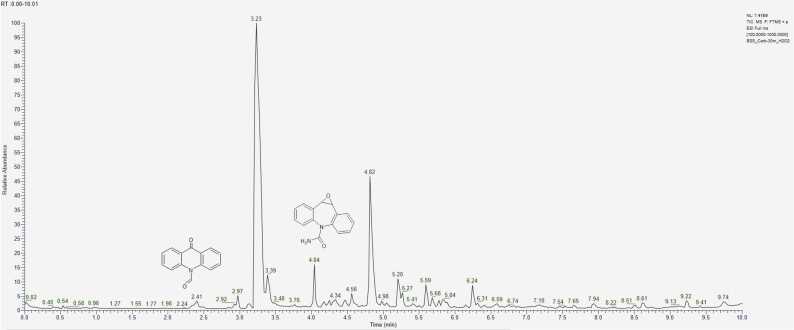

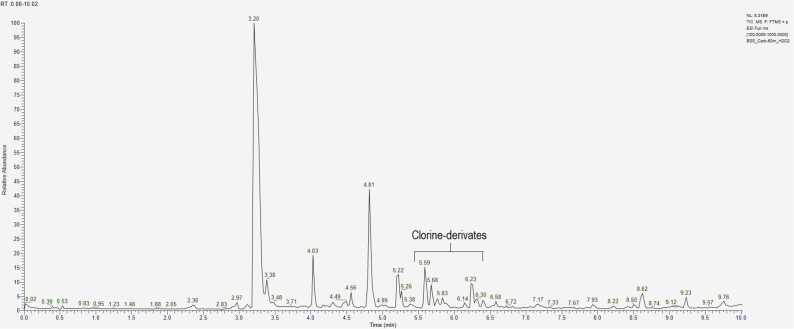
Chromatograms of model samples of CBZ, obtained after interaction with H_2_O_2_ during А) 10 min, B) 30 min, C) 60 min.

Analysing chromatograms obtained after the experiment involving CBZ interaction with hydrogen peroxide H_2_O_2_ over 10, 30, and 60 minutes (Fig. 4), it is evident that the optimal degradation time for accumulation of products lies within the 20–30 minute interval during oxidation. Beyond this time, the signal intensity changes insignificantly. A similar pattern is observed with other aging agents.

Due to the selected mobile phase gradient, the substances of interest elute between 2 and 10 minutes. Therefore, signals beyond 10 minutes do not hold significant analytical importance in this experiment.

The greatest variety of signals was observed during the oxidation of CBZ using a 3 % H_2_O_2_ solution, with the most active transformation being the conversion of CBZ to acridine (AI) and its derivatives (t_R_ = 3.2 – 3.4 min) (Table 7). Additionally, acrid-9-one ([M+H]^+^ = 196.0756) emerged as one of the primary degradation products during oxidation, characterized by an intense peak at a retention time of 4.83 min. Between 10 and 30 minutes, a sharp decrease in the concentration of CBZ in the sample indicates that it has been more completely converted into degradation products. Although CBZ continues to be present in its unmodified form. On the chromatogram (Fig. 4 (B)), there is an increase in peak intensity at a retention time of 4.04 min, which corresponds to degradation products having an ion mass [M+H]^+^ = 253.0971. This mass corresponds to several possible structural formulas, but CBZ-EP is hypothesized to contribute significantly to this signal, as one of the main CBZ metabolites.

**Table 7:** Relative contents of CBZ and its main degradation products (%) under oxidation conditions, calculated by the internal normalization method.

t_R_, min	3.2	4.0	4.8	5.2
Exposure to degradation, min	**AI**	**CBZ-EP**	**acrid−9-one**	**CBZ**
10	45.4	5.0	22.1	27.5
30	58.8	8.7	26.9	5.7
60	58.3	10.6	24.0	7.1

In the experimental conditions, a compound elutes first at t_R_ = 2.38 min with an ion mass [M+H]_+_ = 224.07061, corresponding to a compound with the empirical formula C_14_H_9_NO_2_. At t_R_ = 2.97 min, a signal is formed by an ion with *m/z* = 210.0912, corresponding to a substance with the molecular formula C_14_H_11_NO. Further discussion on the separation of isomeric compounds will be addressed below.

Chlorine-containing degradation products elute after 5.5 minutes (Fig. 4C), and their quantitative content increases with the reaction time. Their formation is likely associated with the addition of hydrochloric acid during the sample preparation stage to adjust the pH, explaining their presence not only in samples exposed to acidic conditions.

This detailed analysis highlights the complexity of degradation pathways and the variety of degradation products formed under different experimental conditions, essential for forensic and toxicological analyses of CBZ in biological samples.

When analysing chromatograms obtained from the interaction of CBZ with 18 % hydrochloric acid, it was found that CBZ undergoes minimal degradation under these conditions, with iminostilbene (IM) being the primary degradation product (Appendix, Fig. S1; Table 8).

**Table 8:** Relative contents of CBZ and its main degradation products (%) under acidic conditions, calculated by the internal normalization method.

t_R_, min	3.2	3.4	4.8	5.2	6.9
Exposure to degradation, min	**AI**	**acridine−9-carbaldehyde**	**acrid−9-one**	**CBZ**	**IM**
10	9.2	7.8	10.3	72.7	0.0
30	7.3	14.6	3.2	43.7	31.3
60	8.0	3.5	8.8	44.1	35.5

CBZ undergoes conversion to IM by elimination of the CONH_2_ group fragment. This process occurs during aging, metabolism, and thermal degradation in the ionization source (400ºC). Acridine and its derivatives are also formed, but in smaller quantities. Despite the presence of HCl, the content of chlorinated derivatives is not significant, even compared to oxidative aging.

In an alkaline medium, the formation of the smallest amount of diverse degradation products for CBZ was observed. In the chromatograms (Appendix, Fig. S2), obtained from the interaction of CBZ with 0.1 M NaOH, there is an intense peak of CBZ along with 10,11-dihydrocarbamazepine, formed by hydrogenation of CBZ. The primary product of alkaline degradation can be identified as IM. Acridine is also formed, but to a lesser extent (Table 9).

**Table 9:** Relative contents of CBZ and its main degradation products (%) under basic conditions, calculated by the internal normalization method.

t_R_, min	3.2	5.2	6.9
Exposure to degradation, min	**AI**	**CBZ**	**IM**
10	0.7	93.4	5.9
30	2.5	89.8	7.7
60	2.8	86.6	10.6

**Fig. 5:**
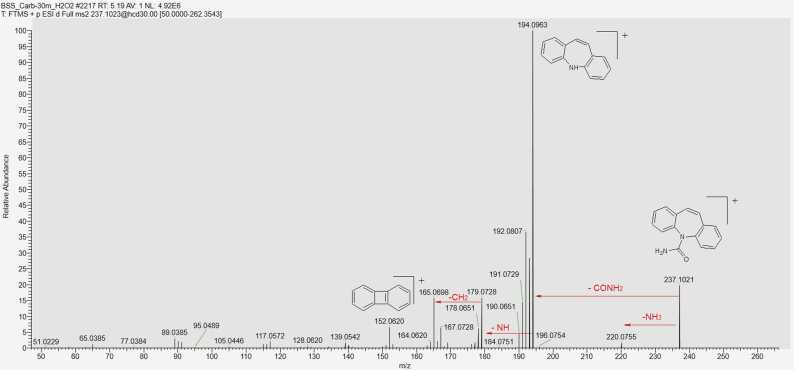
MS/MS spectrum of CBZ.

**Fig. 6:**
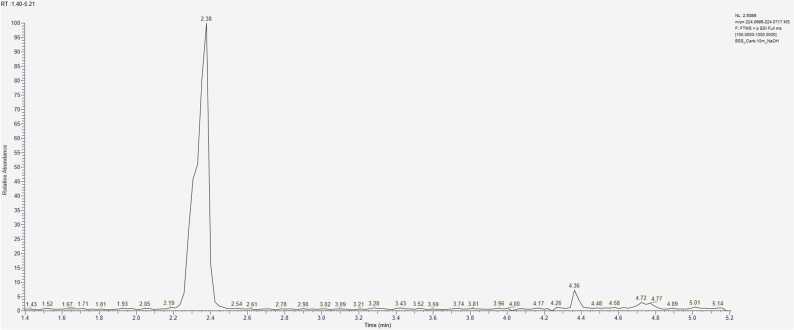
Chromatogram for the [M+H]^+^ = 224,0706 ion.

**Fig. 7:**
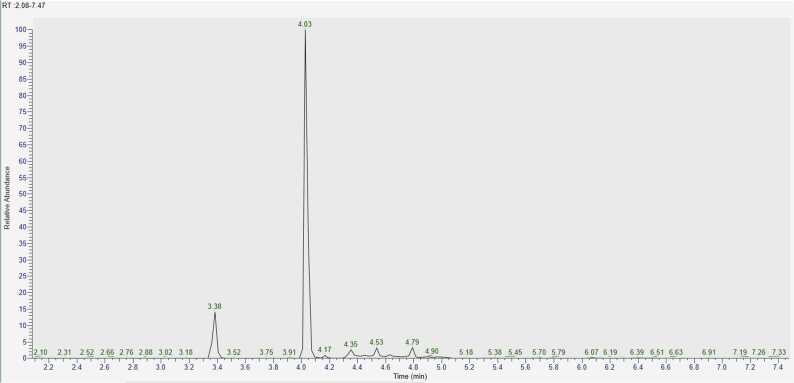
The fragment of chromatogram of CBZ for the *m/z* = 253,09715 ion, obtained after exposure to HCl for 60 min.

**Fig. 8:**
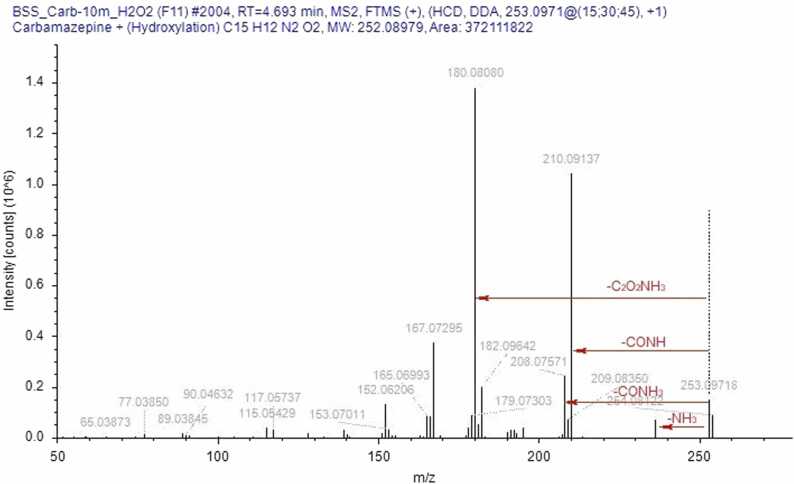
MS/MS spectrum of *m/z* = 253,09718 ion fragmentation.

CBZ in its unmodified form was present in all samples, indicating its incomplete degradation under our experimental conditions. In identifying the degradation products, understanding the fragmentation pathways of CBZ and its derivatives played a key role (Fig. 5) [23], [24], [25]. Characteristic ions in the fragmentation of CBZ include 194.0964, corresponding to IM and formed by the cleavage of the CONH_2_ group; 220.0757, resulting from the loss of ammonia; and 179.0730, formed by the cleavage of the NH-CONH_2_ fragment. The sequential cleavage of another methylene group forms a fragment corresponding to the ion with *m/z* = 165.0698. The ion 152.0620 is formed by rearrangement, resulting in the formation of a new four-membered ring. The molecular ion [M+H]_+_ = 237.10224 is also present in the spectrum, but its intensity is low and does not exceed 20 % under the selected detection conditions. In the mass spectra of CBZ degradation products, recurring fragments characteristic of compounds with a common "core" were observed. For example, the ion [M+H]^+^ = 180.0807, corresponding to acridine (AI), is present with quite high intensity in the fragmentation spectra of all its derivatives (acridine-9-carbaldehyde, 9-oxoacridine-10(9 H)-carbaldehyde, etc.). However, IM derivatives also undergo a rearrangement of the seven-membered ring into a six-membered ring, forming a structure similar to AI. Thus, after hydroxylation, CBZ can undergo further structural changes with the cleavage of the epoxide ring, leading to the diminution of the heterocycle and the formation of 9-oxoacridine-10(9 H)-carbaldehyde, which in turn fragments to IA [25]. Under adverse environmental conditions, the most reactive site in the CBZ molecule is likely the double bond between the carbon atoms C_10_-C_11_. This bond on the central heterocyclic ring can undergo hydroxylation, forming the corresponding hydroxyl derivatives such as 10-OH-CBZ, CBZ-DiOH, or carbamazepine-10,11-epoxide (CBZ-EP). The two external aromatic rings are more inert. In the search for CBZ degradation products in model solutions, some compounds could not be assigned a structural formula due to the absence of a corresponding ion fragmentation spectrum (Table S3 Nos. 7, 9, 16, 18, 21). Based on the mass spectrum containing the ion of interest, we can determine the exact mass of the compound using high-resolution mass spectrometry. By analysing the isotopic peak ratios, we can establish the exact elemental composition of the compound and assign it a gross formula. At this point we can speculate on the structure of the compound, but its structure can only be confirmed by analysing the fragmentation spectrum (MS^2^), taking into account the rearrangement and the fragment ions, which can indicate the presence of substituents and refine the molecular structure. When identifying degradation products, we encountered the challenge of determining the structure of isomeric compounds. The difficulty arose when attempting to assign structural formulas to degradation products with the general formula C_14_H_9_NO_2_. In analysing the mass spectra of the ion [M+H]+ = 224.0706, it was necessary to distinguish between acridine-9-carboxylic acid and 9-oxoacridine-10(9 H)-carbaldehyde (Appendix. Table S1 No. 12). Both compounds share a common core, complicating the task. In the fragmentation mass spectrum of the ion *m/z* = 224.0706 taken at t_R_ = 2.38 min, we observe intense signals of ions *m/z* = 196.0757 and 180.0808, corresponding to the sequential cleavage of the (−CO) and oxygen (−O) groups. By correlating structural features with possible fragmentation pathways, we hypothesized that this spectrum belongs to 9-oxoacridine-10(9 H)-carbaldehyde. In the MS/MS spectrum taken at t_R_ = 4.38, an intense peak of ion *m/z* = 206.0601 is observed, formed from the molecular ion by the loss of water (−H_2_O), followed by sequential cleavage of the (−CO) group *m/z* = 178.0651. Thus, we propose that this spectrum corresponds to acridine-9-carboxylic acid. In Fig. 6, the chromatogram for the selected ion [M+H]^+^ = 224.0706 shows that the peak at t_R_ = 2.38 is the most intense, suggesting that the formation of 9-oxoacridine-10(9 H)-carbaldehyde is more favourable during degradation. A large group of degradation products of CBZ includes compounds formed through hydration, epoxidation, and oxidation processes (Appendix. Table S1, No. 17): Carbamazepine-10,11-epoxide (CBZ-EP), carbamazepine-2,3-epoxide, hydroxy-5H-dibenzazepine-5-carboxamide, 10-hydroxy-5H-dibenzazepine-5-carboxamide, 10-oxo-10,11-dihydro-5H-dibenzazepine-5-carboxamide, and 9-formylacridine-10(9 H)-carboxamide. All these compounds share the same empirical formula C_15_H_12_N_2_O_2_ and, of course, ion mass [M+H]^+^ = 253.0972. Consequently, their separation and identification solely through mass spectrometric methods are nearly impossible [26], [27]. Fig. 7 presents a selective chromatogram for the ion *m/z* = 253.0972 obtained during aging in acidic conditions. It is evident that compounds with this protonated ion mass predominantly elute within the time range of 3.3 – 5.0 minutes. The close retention times again confirm the assumption of shared structural characteristics among this group of compounds, yet they exhibit differences in physicochemical properties and represent distinct degradation products. The mass spectrum fragmentation of the ion *m/z* = 253.0972 is depicted in Fig. 8, revealing a structure characteristic of CBZ fragmentation. Therefore, the separation of these isomers is only feasible with specially tailored chromatographic conditions, which may be an area of interest for future research.

**Fig. 9:**
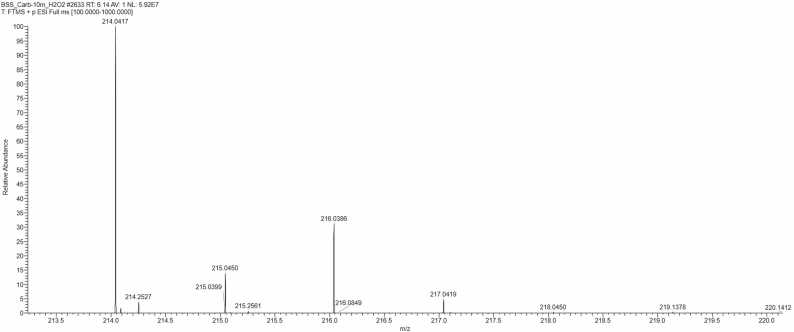
Mass spectrum of isotopic distribution of 9-chloroacridine.

Identification of chlorinated compounds began with analysing the isotopic distribution in mass spectra (MS) and identifying a unique pattern of intensity ratios between peaks A and A+ 2 (3:1), which is characteristic of chlorine atoms in the molecule. With knowledge of the natural abundance of isotopes, the presence of chlorine atoms can be easily detected by the characteristic multiplet signal for this element. Fig. 9 shows the mass spectrum for 9-chloroacridine; the typical isotopic distribution of compounds containing a chlorine atom is evident.

**Fig. 10:**
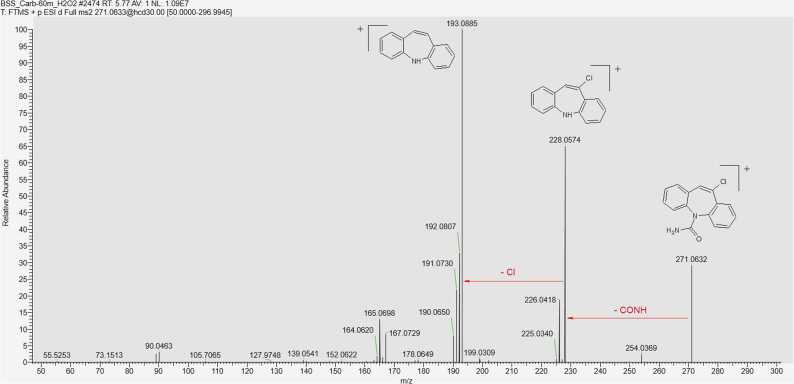
MS/MS spectrum of fragmentation of [M+H]+ = 271,063 ion, corresponding to 10-Cl-CBZ.

Fragmentation analysis of the chlorine-containing degradation products shows the loss of one chlorine atom (loss of *m/z* = 34.968), while maintaining a general fragmentation pattern characteristic of CBZ. Fig. 10 shows the fragmentation spectrum of 10-chlorocarbazepine (10-Cl-CBZ) (see Appendix Table S3, No. 20).

During the study, 22 degradation products of CBZ were identified in model solutions subjected to artificial aging using 18 % HCl, 0.1 M NaOH, and 3 % H_2_O_2_. It was found that oxidation of CBZ leads to significant molecule degradation, indicating its instability under oxidative conditions. However, all identified degradation products, except iminodibenzyl, were present in all model solutions, indicating the overall instability of CBZ in adverse environmental conditions and its tendency towards chemical modifications and degradation.

### 4.5 Analysis of samples of human liver tissue

Forensic samples of human liver autopsy evidence provided by the Federal State Budgetary Institution "Research Centre for Medical Genetics" of the Ministry of Health of Russia were analysed under the same conditions as the model samples. Analysis of the two forensic samples containing CBZ (FSE_1, FSE_2) revealed 2 of the 22 degradation products described above (Table 10).

**Table 10:** Compounds (CBZ and products of its degradation) found in the human liver samples.

Iminostilben (IM) were identified in the two forensic samples both when analysed immediately and after air aging for 1, 3, 5 and 8 weeks. It can be hypothesized that IM may have formed in the tissue through degradation processes as well as natural metabolism. The second detected degradation product had an ion [M+H]^+^= 253.0973, with a mass corresponding to the empirical formula C_15_H_12_N_2_O_2_ and a series of structures described previously. Based on available literature sources, we speculate that the ion with this *m/z* value may correspond to carbamazepine-10,11-epoxide (CBZ-EP), the most expected metabolite of CBZ. CBZ-EP was also present in intact, unpreserved liver samples, indicating its metabolic origin. However, when assessing the relative content of CBZ and CBZ-EP in samples exposed to environmental conditions for 8 weeks (Table 11), we observe a gradual increase in the percentage content of CBZ-EP over time, indicating ongoing degradation of CBZ.

**Table 11:** Relative content of CBZ и CBZ-EP (%) in the liver samples, calculated by internal normalization method.

	FSE_1		FSE_2	
Weeks	**CBZ**	**CBZ-EP**	**CBZ**	**CBZ-EP**
0	98.9	1.1	99.2	0.8
1	98.8	1.2	98.7	1.3
3	98.2	1.8	98.3	1.7
5	98.0	2.0	98.1	1.9
8	97.2	2.8	97.8	2.2

During the analysis of human liver samples (FSE_1, FSE_2), compounds unrelated to the metabolism and degradation products of CBZ were detected. Identification was performed using the TraceFinder. Forensic software (Thermo Scientific™), designed for chemical-toxicological and forensic analysis, along with the NIST Mass Spectral Search Program database and a database of narcotic and toxic substances provided by the Federal State Budgetary Institution "RCME" of the Ministry of Health of Russia.

**Fig. 11:**
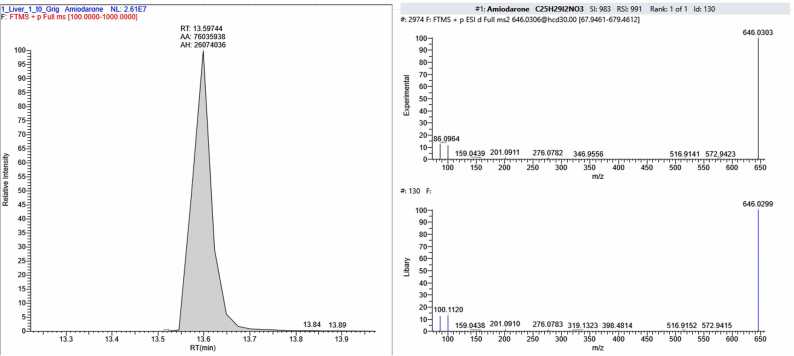
The section of the chromatogram, MS spectrum of fragmentation and library spectrum of amiodarone, found the sample FSE_1.

In the human liver sample FSE_1, in addition to CBZ, amiodarone was detected (Fig. 11). This compound is a part of the antiarrhythmic drug of the same name and possesses antianginal properties.

**Fig. 12:**
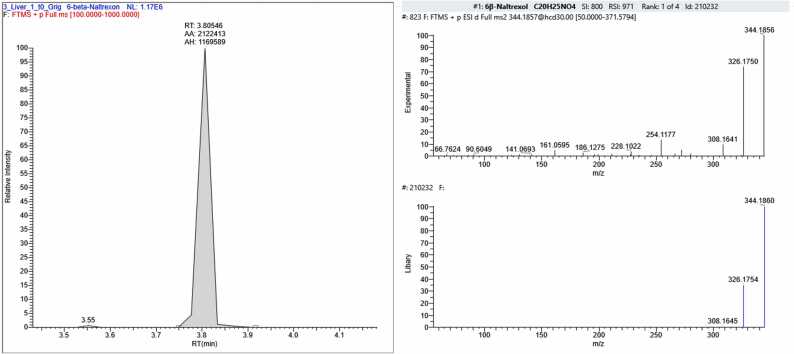

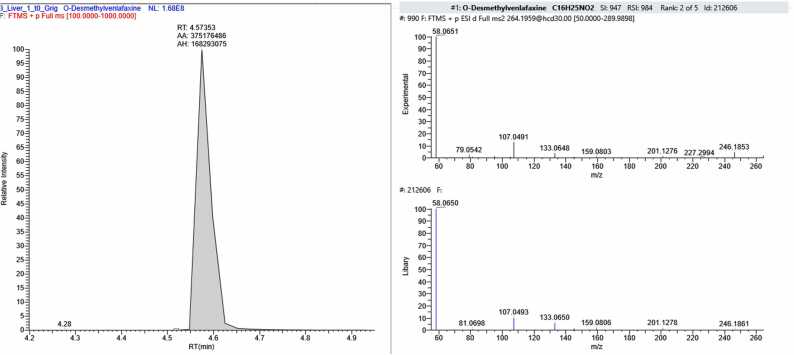

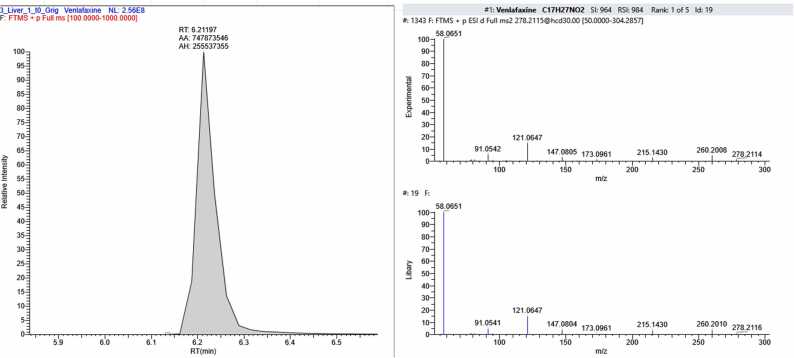

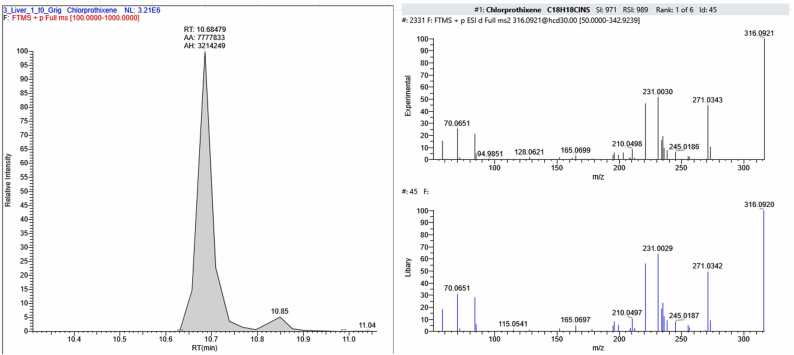
The section of the chromatogram, MS spectrum of fragmentation and library spectrum of compounds (6-beta-naltrexone (С_20_Н_25_NO_4_), Venlafaxine (С_17_H_27_NO_2_), Dimethylvenflaxine (С_16_H_25_NO_2_), Chlorprothixene (С_18_H_18_ClNS) found the sample FSE_2.

In sample FSE_2, four compounds were found in addition to CBZ: 6-beta-naltrexone (a metabolite of naltrexone, an opioid receptor antagonist used for opioid and alcohol dependence), venlafaxine – (an antidepressant belonging to the group of selective serotonin and norepinephrine reuptake inhibitors, prescribed for depressive and anxiety disorders), its metabolite desmethylvenlafaxine and chlorprothixene (a neuroleptic with sedative and antipsychotic effects) (Fig. 12).

## 5 Conclusions

A methodology for qualitative and quantitative determination of carbamazepine (CBZ) in human liver tissue using HPLC-HRMS on a Vanquish chromatographic system (Thermo Scientific™, USA) coupled with an Orbitrap Exploris 120 mass spectrometer (Thermo Scientific™, USA) has been developed. Validation of the developed method has been conducted, and key analytical characteristics have been determined. The limit of detection (LOD) is 1 ng/g, the lower limit of quantitation (LLOQ) or minimum detectable concentration is 5 ng/g (Fig. S7). The working range of calibration covers concentrations from 5 to 2000 ng/g.

Method accuracy meets the requirements for analytical methods used in forensic chemistry and chemico-toxicological analysis of biological materials, with deviations not exceeding ± 20 % for all control concentration levels. Signal recovery evaluation showed that the analyte signal in samples does not exceed 20 % of the LLOQ signal level, and the internal standard signal in samples does not exceed 5 % of the background signal. The developed method complies with the validation guidelines of the Federal State Budgetary Institution "RCM&E" of the Ministry of Health of Russia. Degradation products of carbamazepine in model solutions were investigated under the influence of hydrochloric acid, sodium hydroxide, and hydrogen peroxide oxidation. Twenty-two degradation products were identified. It was found that the most intensive degradation of carbamazepine with the formation of various degradation products occurs during oxidation in an acidic solution adjusted to pH= 1–2 with 3 % hydrogen peroxide. The stability of carbamazepine in human liver tissues under environmental conditions over different periods of time was investigated. The experiment showed that CBZ tends to degrade under environmental conditions. The maximum decrease in concentration was observed during the first week of storage (on average by 20 %), with a subsequent approximate halving of concentration over 8 weeks. This relationship allows estimating the possible lethal dose in poisoning and the time of death.

The presented data, when carrying out forensic medical examination, will allow to estimate the level of toxicant concentration in biological material shortly before death. This is relevant in case of finding a corpse 1–2 months after death.

Analysis of two post-mortem forensic liver samples provided by the Federal State Budgetary Institution "RCM&E" of the Ministry of Health of Russia (FSE_1 and FSE_2) revealed CBZ concentrations corresponding to toxic levels in tissue. The measured concentration was 3150 ± 280 ng/g for FSE_1 and 7880 ± 820 ng/g for FSE_2. The upper limits of therapeutic concentration ranges were exceeded by 1.5 and 3.9 times, respectively. Based on the analysis of the forensic liver samples (FSE_1 and FSE_2), two out of the 22 CBZ degradation products described in this study were detected. Additionally, amiodarone was found in FSE_1, and four compounds representing pharmaceutical substances or their metabolites were identified in FSE_2. Therefore, combined poisoning could have been a possible cause of death.

## CRediT authorship contribution statement

**Barge Alessandro:** Writing – review & editing, Validation. **Nosyrev Aleksander:** Funding acquisition. **Barsegyan Samvel:** Writing – review & editing, Supervision, Resources, Methodology. **Akimova Valeriya:** Writing – review & editing, Supervision, Data curation. **Gandlevskiy Nikita:** Writing – review & editing, Formal analysis, Data curation, Conceptualization. **Shirokova Ekaterina:** Investigation, Formal analysis, Data curation. **Pirogov Andrei:** Writing – original draft, Project administration, Conceptualization.

## Declaration of Competing Interest

The authors declare that they have no known competing financial interests or personal relationships that could have appeared to influence the work reported in this paper.

## Data Availability

Data will be made available on request.

## Footnotes

Appendix A Supplementary data associated with this article can be found in the online version at doi:10.1016/j.toxrep.2025.101923.
