# Supplementary material for: Highly-sensitive quantification of carbamazepine and identification of its degradation and metabolism products in human liver by high performance liquid chromatography – High resolution mass spectrometry
Source: Toxicol Rep. 2025 Jan 27;14:101923. doi: 10.1016/j.toxrep.2025.101923 (PMC11803175; doi:10.1016/j.toxrep.2025.101923)
Supplement: Supplementary file 1 — Supplementary material [file mmc1.docx]

# **Supplementary**

Table S1. Results of quantification of CBZ in sample of cadaveric human liver FSE_1, Р=0.95, n=5, n=4

| weeks | Sample.repetition | С, ng/g | С average, ng/g | RSD, % | Absolute error |
| --- | --- | --- | --- | --- | --- |
| 0 | 1.1 | 2927.482 | 3149.721 | 7.10 | 277.673 |
|  | 1.2 | 2927.941 |  |  |  |
|  | 1.3 | 3152.999 |  |  |  |
|  | 2.1 | 3323.578 |  |  |  |
|  | 2.2 | 3416.606 |  |  |  |
| 1 | 1.1 | 2059.527 | 2279.075 | 6.89 | 249.754 |
|  | 1.2 | 2276.893 |  |  |  |
|  | 2.1 | 2413.59 |  |  |  |
|  | 2.2 | 2366.289 |  |  |  |
| 3 | 1.1 | 1766.598 | 1652.887 | 11.69 | 307.475 |
|  | 1.2 | 1858.501 |  |  |  |
|  | 2.1 | 1547.19 |  |  |  |
|  | 2.2 | 1439.26 |  |  |  |
| 5 | 1.1 | 1629.096 | 1555.564 | 7.94 | 196.447 |
|  | 1.2 | 1684.123 |  |  |  |
|  | 2.1 | 1413.472 |  |  |  |
|  | 2.2 | 1495.564 |  |  |  |
| 8 | 1.1 | 1465.726 | 1352.286 | 10.93 | 235.131 |
|  | 1.2 | 1390.263 |  |  |  |
|  | 2.1 | 1135.632 |  |  |  |
|  | 2.2 | 1417.522 |  |  |  |

Table S2. Results of quantification of CBZ in expert sample of human liver FSE_2. Р=0.95, n=5, n=4

| weeks | Sample.repetition | С, ng/g | С average, ng/g | RSD % | Absolute error |
| --- | --- | --- | --- | --- | --- |
| 0 | 1.1 | 7342.765 | 7884.382 | 8.41 | 823.326 |
|  | 1.2 | 7441.83 |  |  |  |
|  | 1.3 | 8119.307 |  |  |  |
|  | 2.1 | 8942.247 |  |  |  |
|  | 2.2 | 7575.763 |  |  |  |
| 1 | 1.1 | 7119.307 | 6698.242 | 10.39 | 1107.415 |
|  | 1.2 | 7299.488 |  |  |  |
|  | 2.1 | 5744.539 |  |  |  |
|  | 2.2 | 6629.635 |  |  |  |
| 3 | 1.1 | 5439.299 | 6057.292 | 14.76 | 1422,689 |
|  | 1.2 | 5269.303 |  |  |  |
|  | 2.1 | 7211.354 |  |  |  |
|  | 2.2 | 6309.211 |  |  |  |
| 5 | 1.1 | 5979.963 | 5657.728 | 10.55 | 950.095 |
|  | 1.2 | 6236.459 |  |  |  |
|  | 2.1 | 4871.913 |  |  |  |
|  | 2.2 | 5542.576 |  |  |  |
| 8 | 1.1 | 5130.541 | 4564.364 | 9.69 | 703.976 |
|  | 1.2 | 4680.36 |  |  |  |
|  | 2.1 | 4324.503 |  |  |  |
|  | 2.2 | 4122.052 |  |  |  |

| Table S3. Estimated structural formulas, ion masses and mass spectra of carbamazepine degradation products obtained during artificial aging in acidic and alkaline environments, as well as during oxidation. | | | | |
| --- | --- | --- | --- | --- |
| № | Brutto formula, name, structure | Monoisotopic mass and ion masses, Da | Retention time, min | Mass spectra of fragmentation  (*if it has been recorded)* |
| 0 | C_15_H_12_N_2_O  carbamazepine (CBZ)  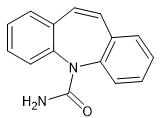 | [M] = 236.0950  [M+H]^+^ = 237.1022  [M-H]^-^ = 235.0877 | 5.2-5.4 | 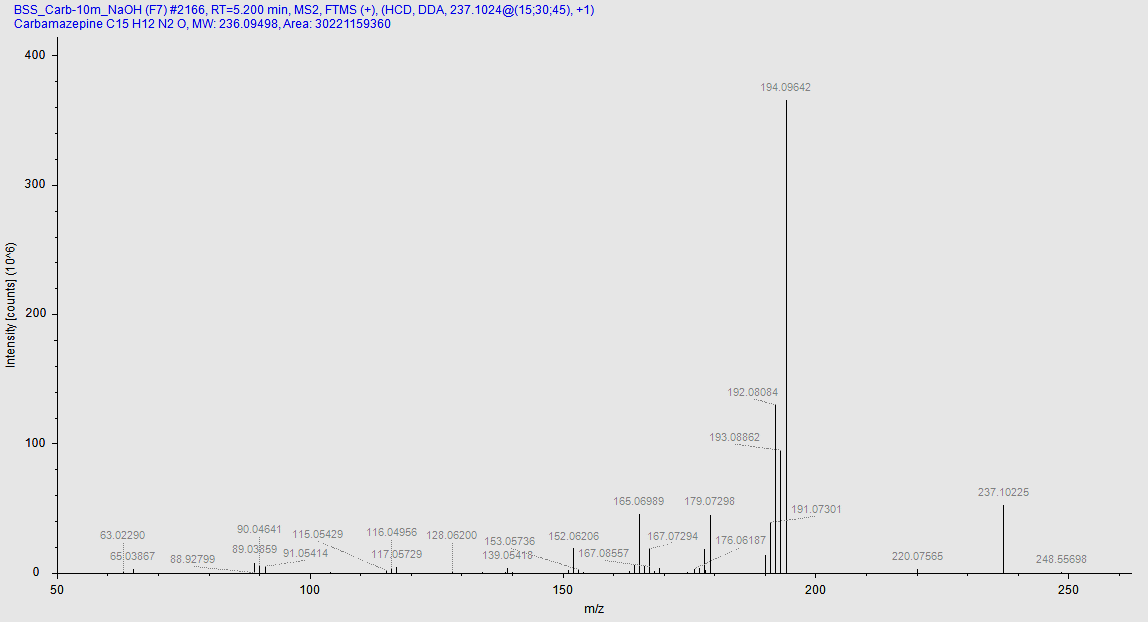 |
| 1 | C_14_H_9_NO  acridine-9-carbaldehyde  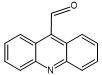 | [M] = 207.0684  [M+H]^+^ = 208.0757  [M-H]^-^ =206.0611 | 3.4-4.3 | 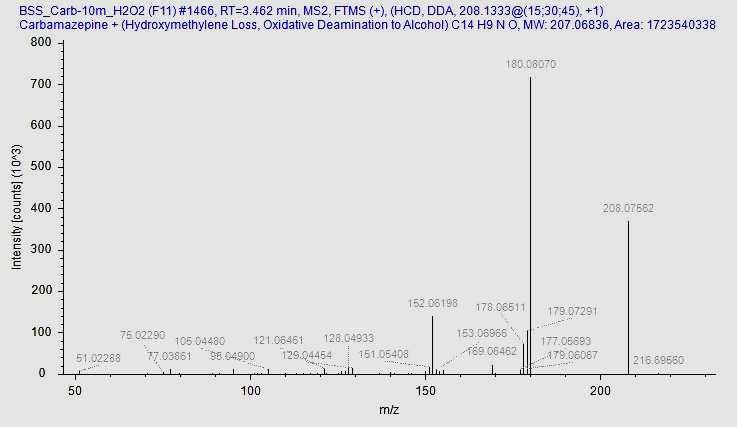 |
| 2 | C_13_H_9_N  acridine (AI)  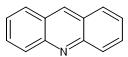 | [M] = 179.0735  [M+H] ^+^ = 180.0808  [M-H]^-^ =178.0662 | 3.2-3.3 | 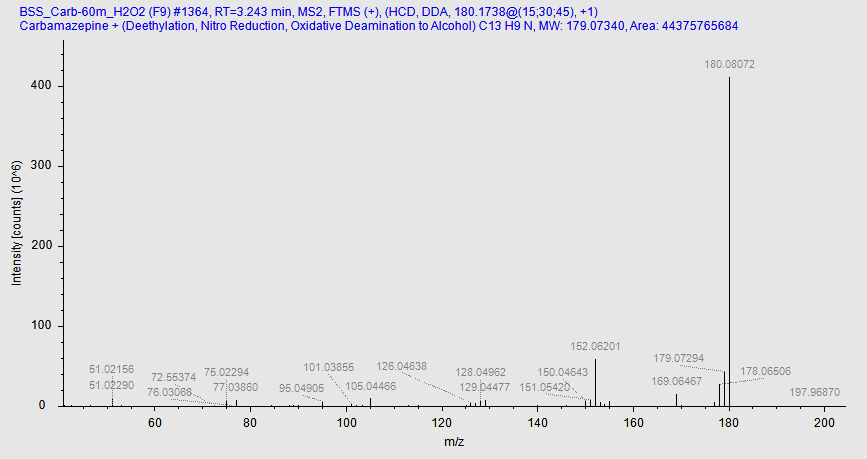 |
| 3 | C_13_H_9_NO  acrid-9-one  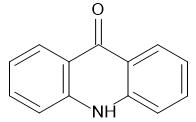 | [M] = 195.0684  [M+H] ^+^ = 196.0757  [M-H] ^-^ = 194.0611 | 4.81-4.83 | MS1 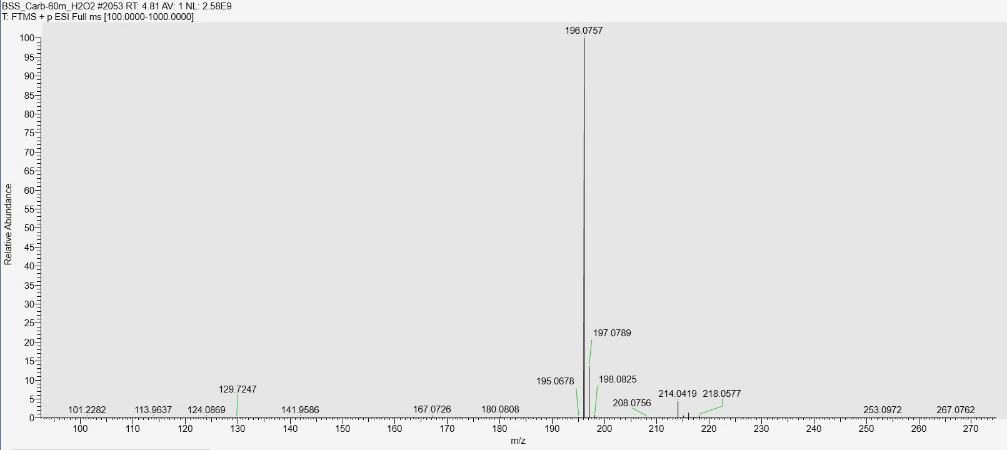  MS2(-) 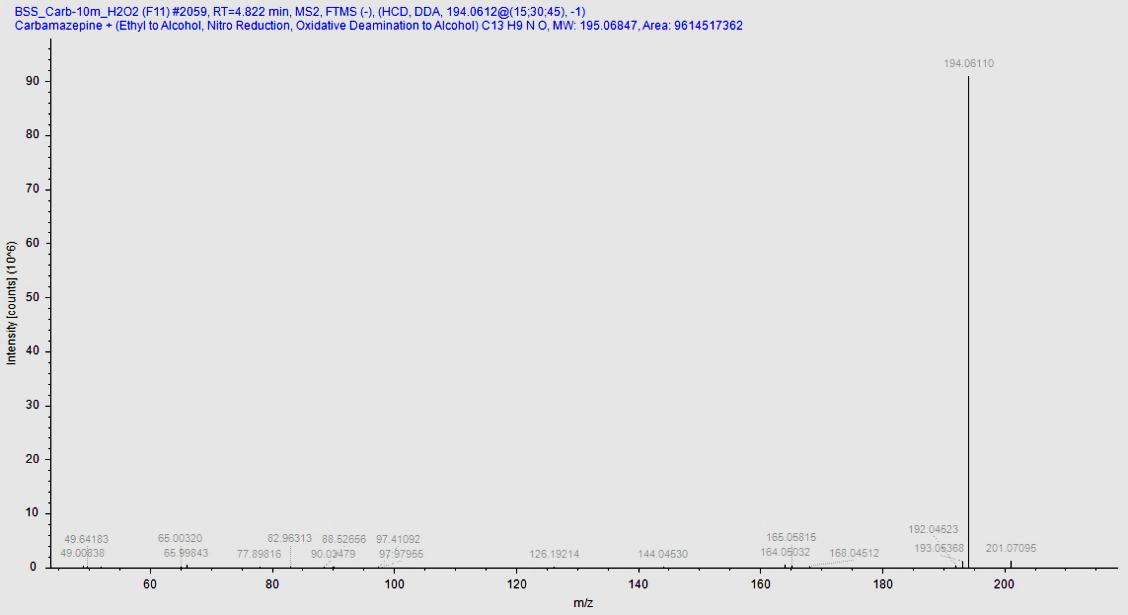 |
| 4 | C_14_H_11_N  iminostilbene (IM)  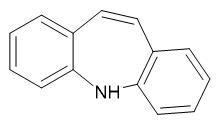 | [M] = 193.0891  [M+H] ^+^ = 194.0964  [M-H] ^-^ = 192.0819 | 6.99 | 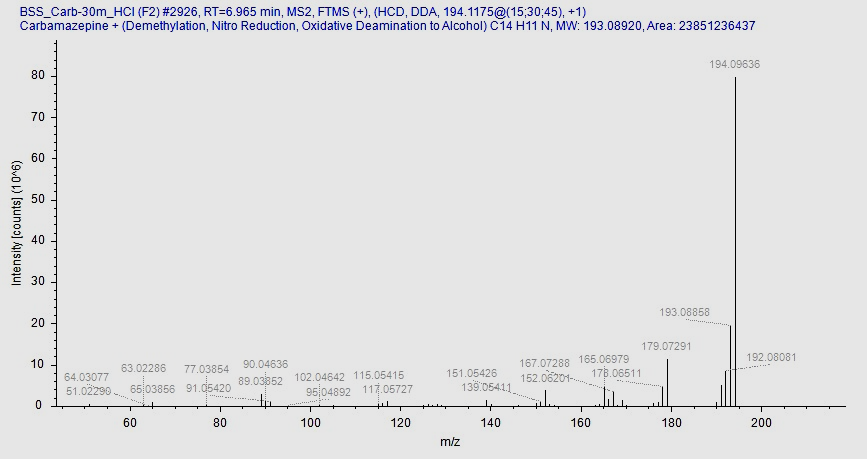 |
| 5 | C_14_H_13_N  iminodibenzyl  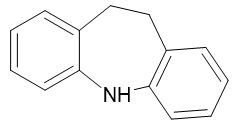 | [M] = 195.1048  [M+H] ^+^ = 196.1121  [M-H] ^-^ = 194.0975 | 7.4 | 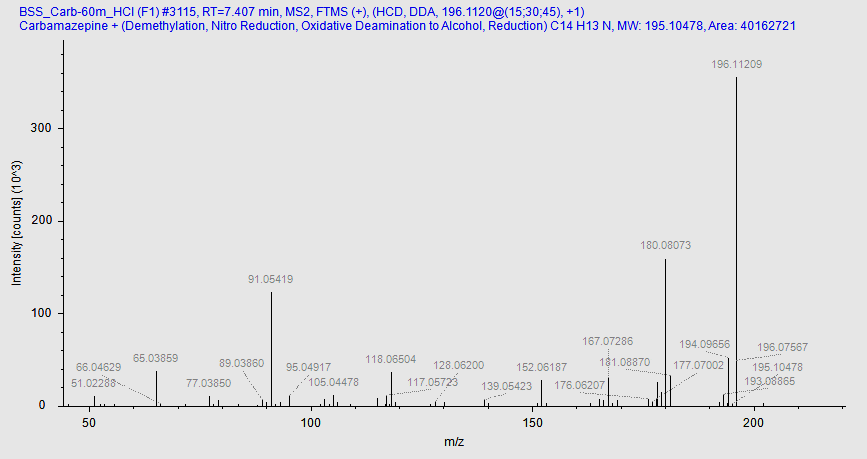 |
| 6 | C_15_H_14_N_2_O  10,11-dihydrocarbamazepine  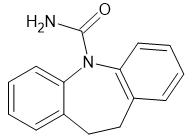 | [M] = 238.1101  [M+H] ^+^ = 239.1179  [M-H] ^-^ = 237.1033 | 5.2-5.3 | 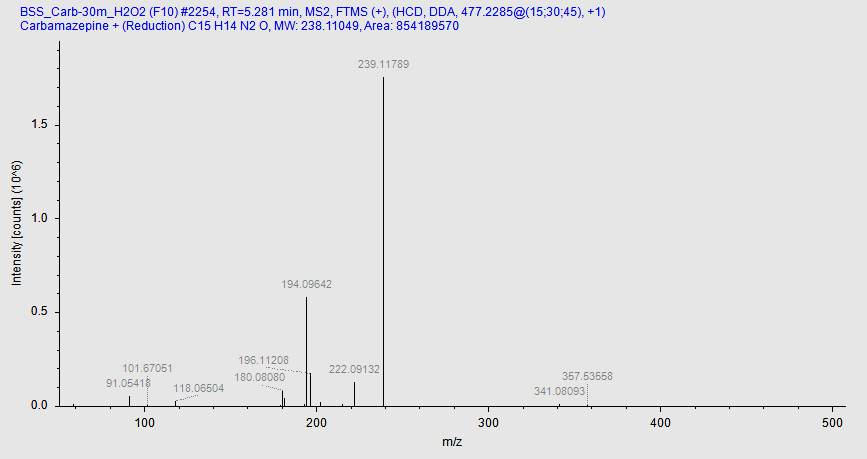 |
| 7 | С_15_H_14_N_2_O_2_  *MS2 spectrum was not recorded* | [M] = 254.1055  [M+H] ^+^ = 255.1128  [M-H] ^-^ = 253.0983 | 4.2 | MS1 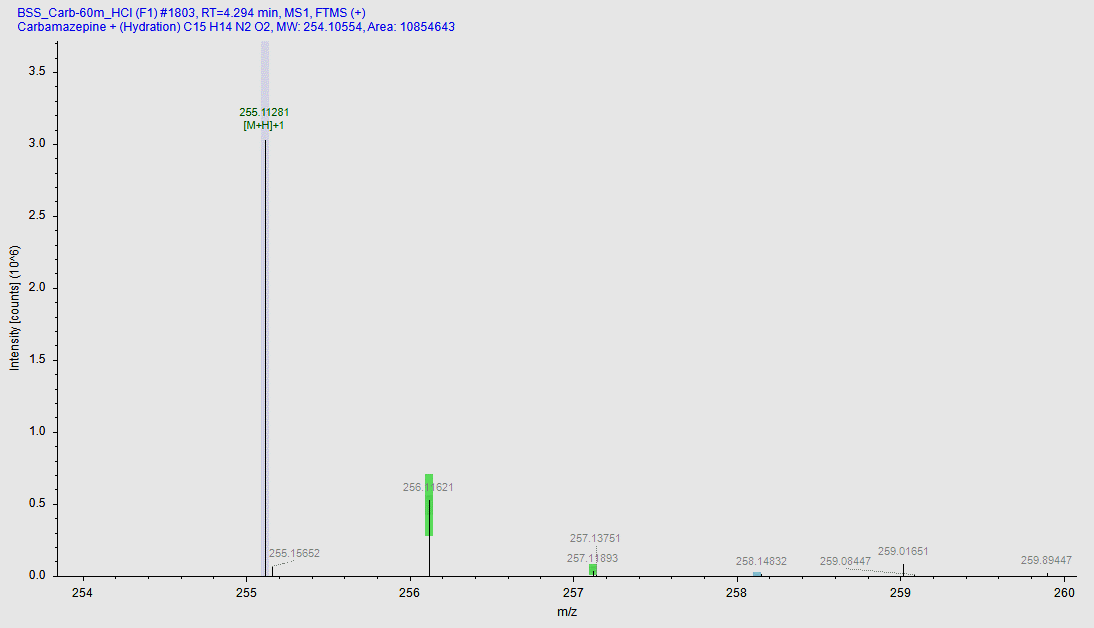 |
| 8 | C_15_H_14_N_2_O_2_  9-(hydroxymethyl) acridine-10(9Н)-carboxamide  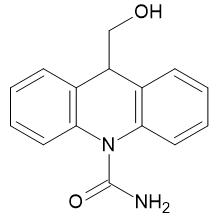 | [M] = 254.1055  [M+H] ^+^ =255.1128  [M-H]^-^= 253.0983 | 6.45 | 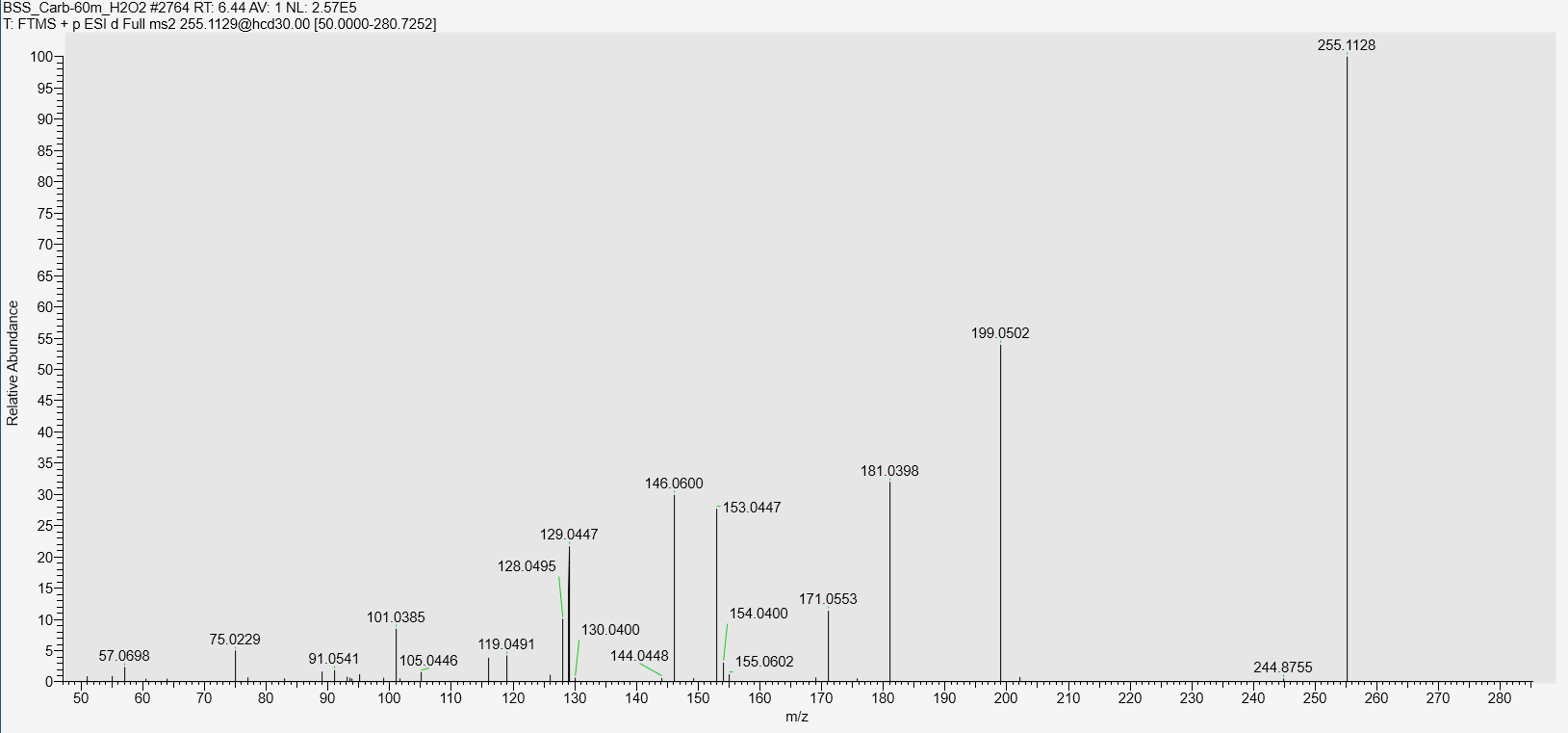 |
| 9 | C_15_H_14_N_2_O_3_  *MS2 spectrum was not recorded* | [M] = 270.1004  [M+H]^+^=271.1077  [M-H] ^-^= 269.0932 | 4.0 | MS1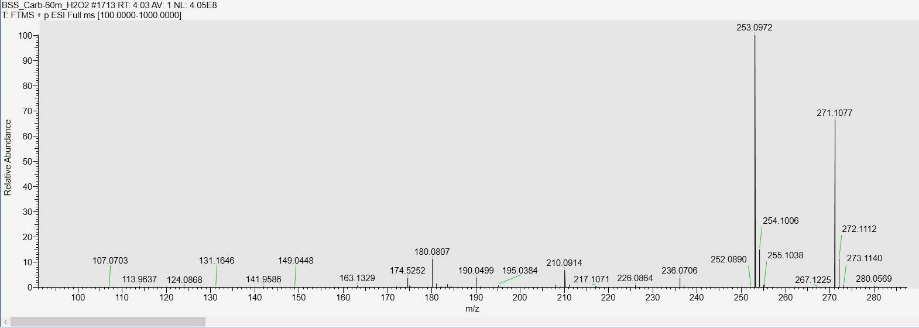 |
| 10 | C_15_H_10_N_2_O_3_  10,11-dioxo- carbamazepine (10,11-diOx-CBZ)  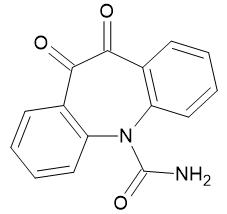 | [M] = 266.0691  [M+H]^+^=267.0764  [M-H]^-^= 265.0619 | 4.3 | 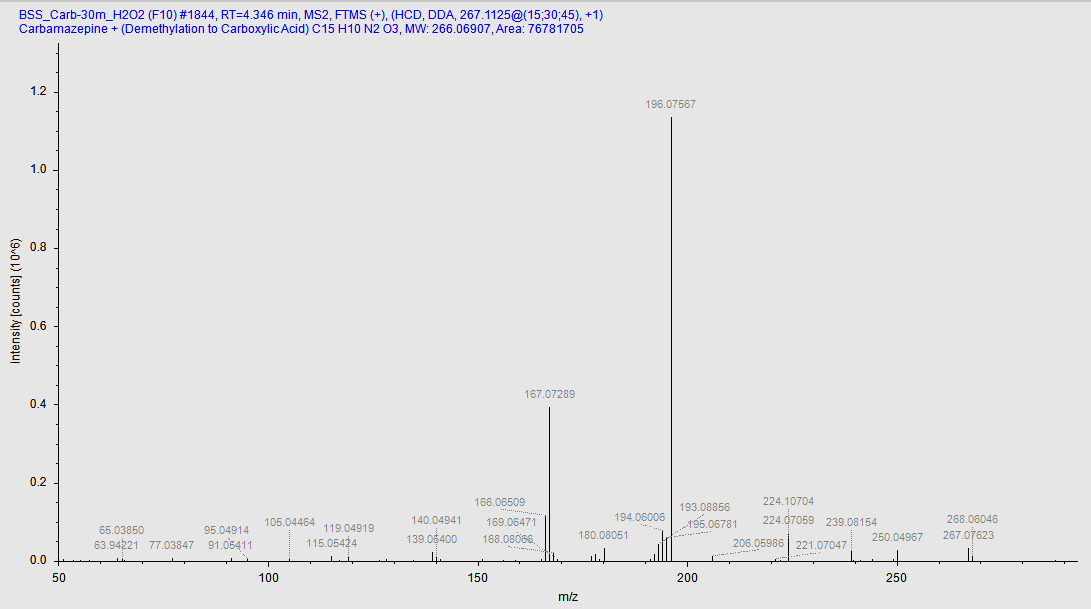 |
| 11 | C_15_H_10_N_2_O_4_  10,11-dioxo-hydrocarbamazepine  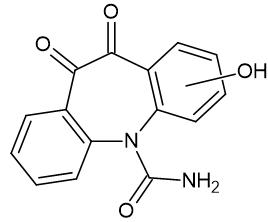 | [M] = 282.0635  [M+H] ^+^ = 283.0713  [M-H] ^-^ = 281.0568 | 4.5 | 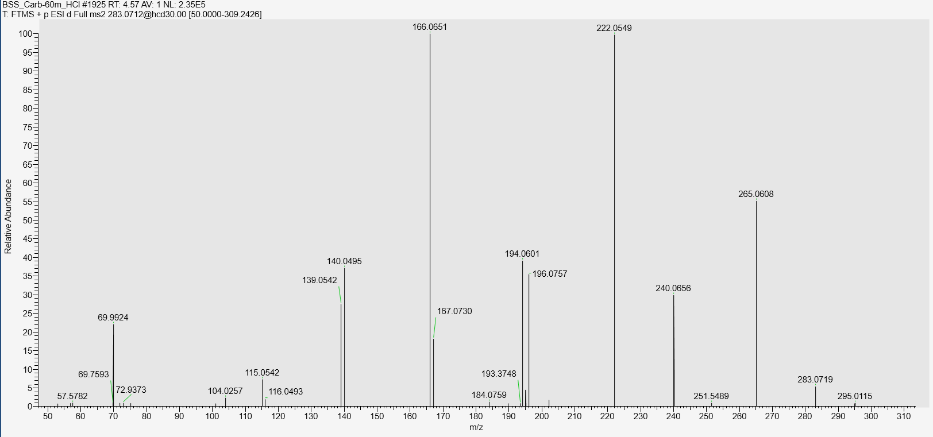 |
| 12 | C_14_H_9_NO_2_  9-oxoacridine-10(9Н)-carbaldehyde  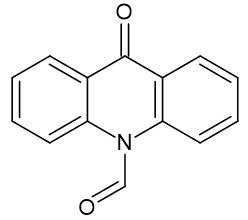 | [M] = 223.0633  [M+H] ^+^ = 224.0706  [M-H] ^-^ = 222.0561 | 2.3 | 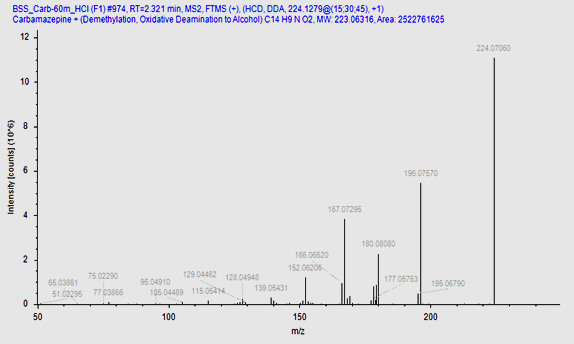 |
| 13 | C_14_H_9_NO_2_  acridine-9-carboxylic acid  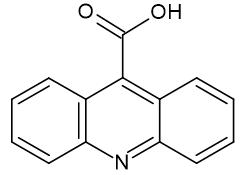 | [M] = 223.0633  [M+H] ^+^ = 224.0706  [M-H] ^-^ = 222.0561 | 4.3 | 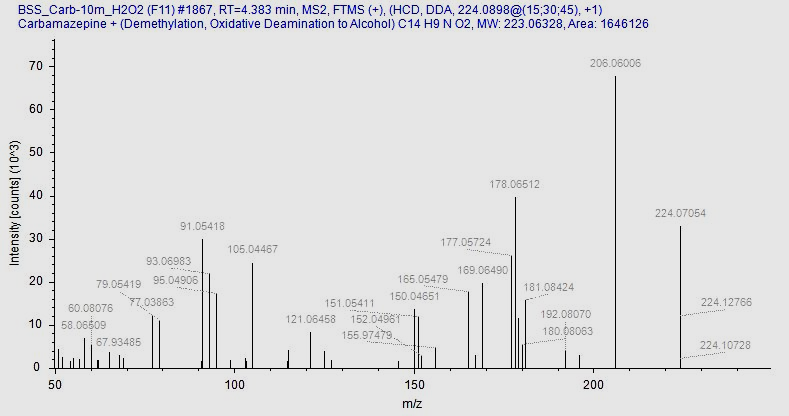 |
| 14 | C_14_H_11_NO  (acridine-9-yl) methanol  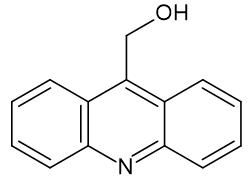 | [M] = 209.0841  [M+H] ^+^ = 210.0913  [M-H] ^-^ = 208.0768 | 4.09 | 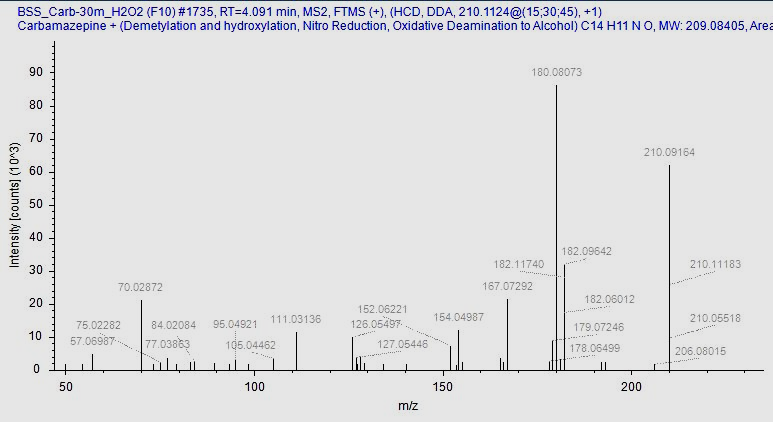 |
| 15 | C_14_H_11_NO  2Н – dibenzoazepine-10-on  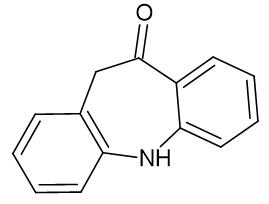  or  5H – dibenzoazepine - 2-ol  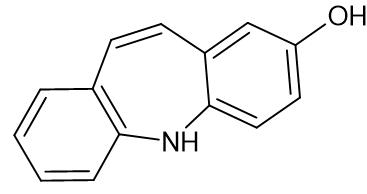 | [M] = 209.0841  [M-H]^+^=210.0913  [M-H] ^-^= 208.0768 | 2.9 | 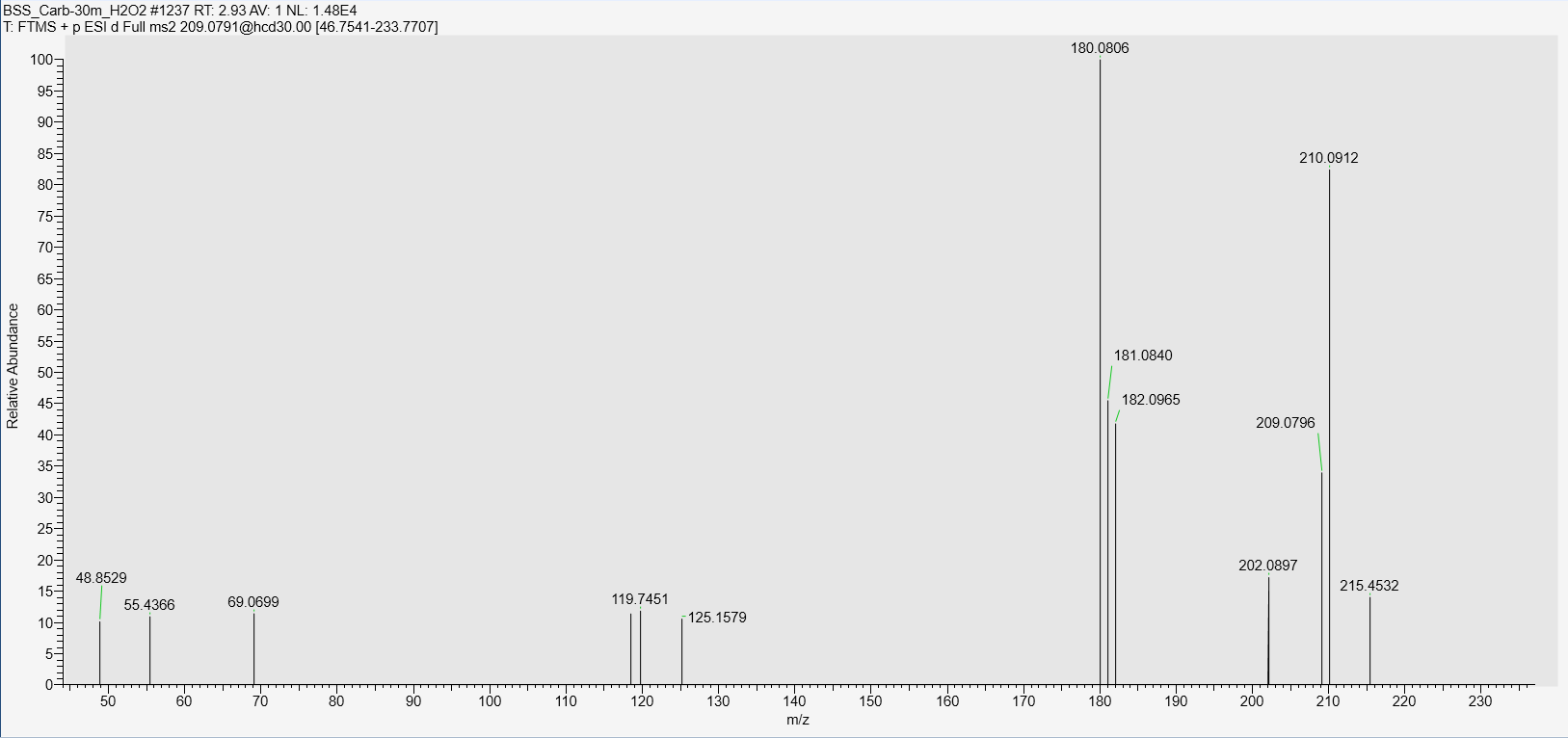 |
| 16 | C_15_H_12_N_2_O_3_  *MS2 spectrum was not recorded* | [M] ^+^ = 268.0848  [M-H]^+^= 269.0921  [M-H ^-^ =267.0775 | 4.46 | 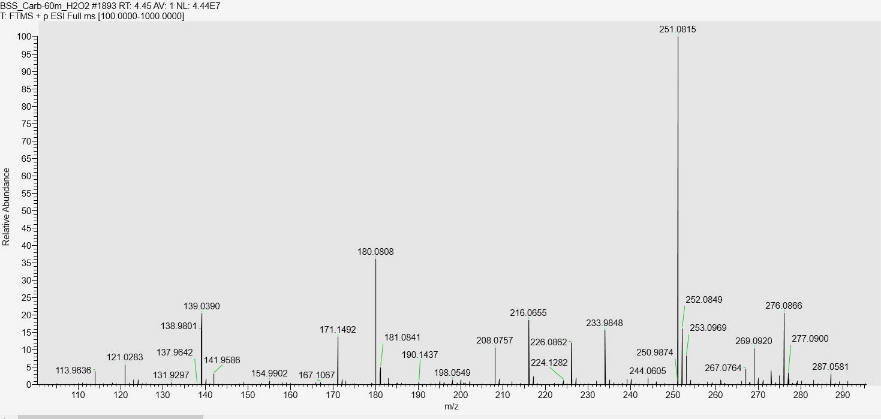 |
| 17 | С_15_H_12_N_2_O_2_  Carbamazepine-10,11-epoxide(CBZ-EP)  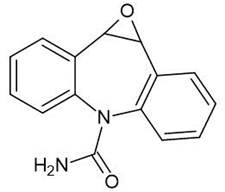  or  hydorxy-5Н -dibenzoazepine -5-carboxamide  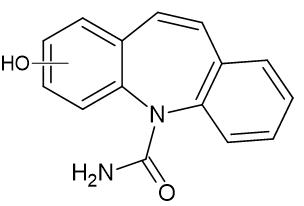  10-hydroxy-5Н-dibenzoazepine-5-carboxyamide  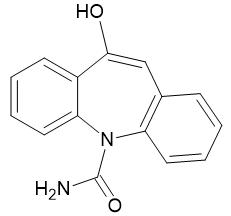  carbamazepine-2,3-epoxide  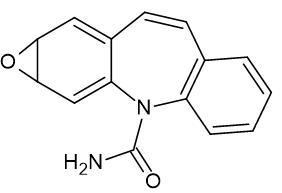  10-oxo-10,11-dihydro-5Н-dibenzoazepine-5-carboxamid  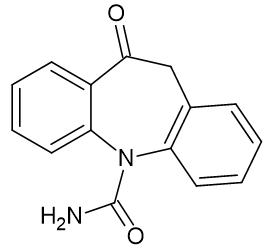  9-formylacridine-10(9Н)-carboxamid  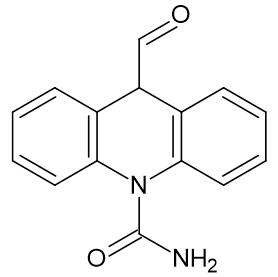 | [M] ^+^ = 252.0893  [M+H]^+^=253.0972  [M-H] ^-^ = 251.0826 | 3.38  4.05  4.36  4.56  4.69  4.75 | 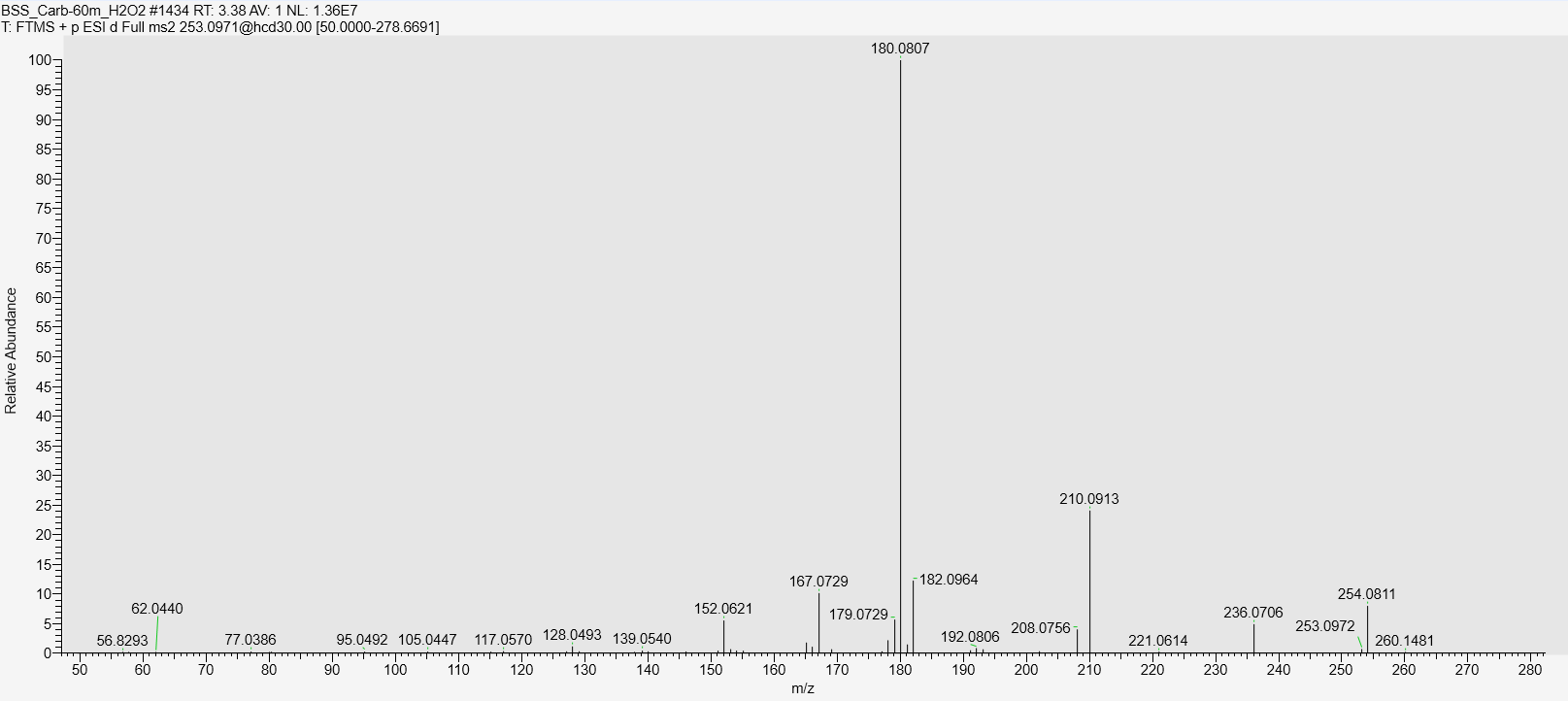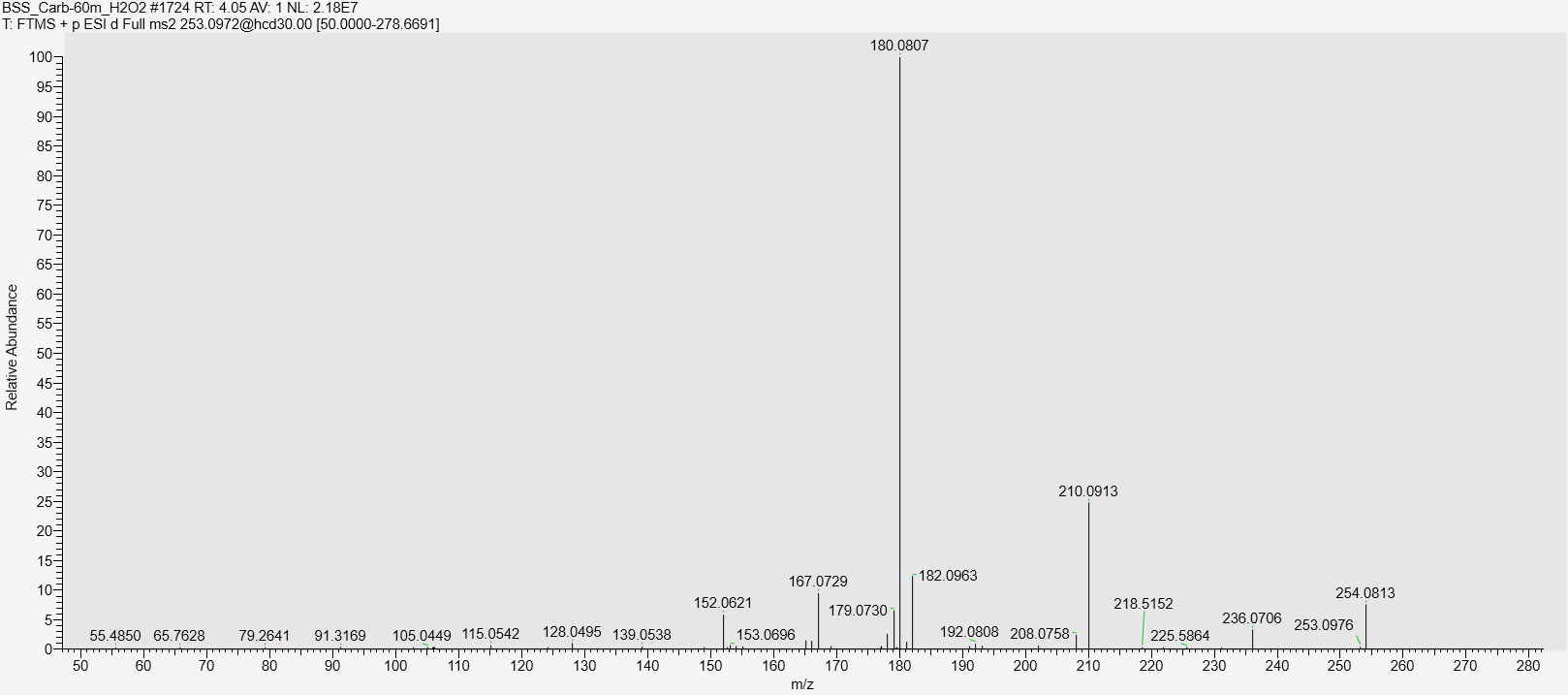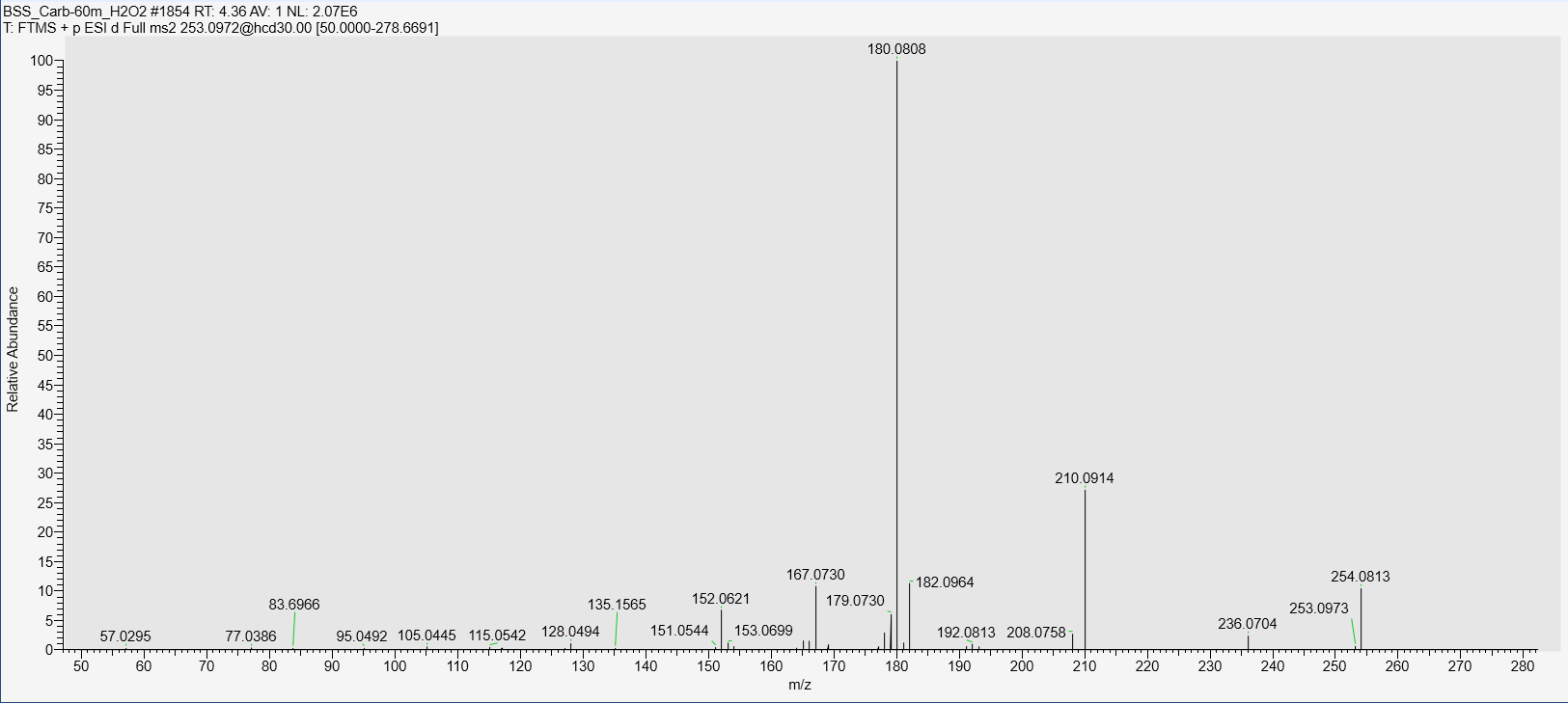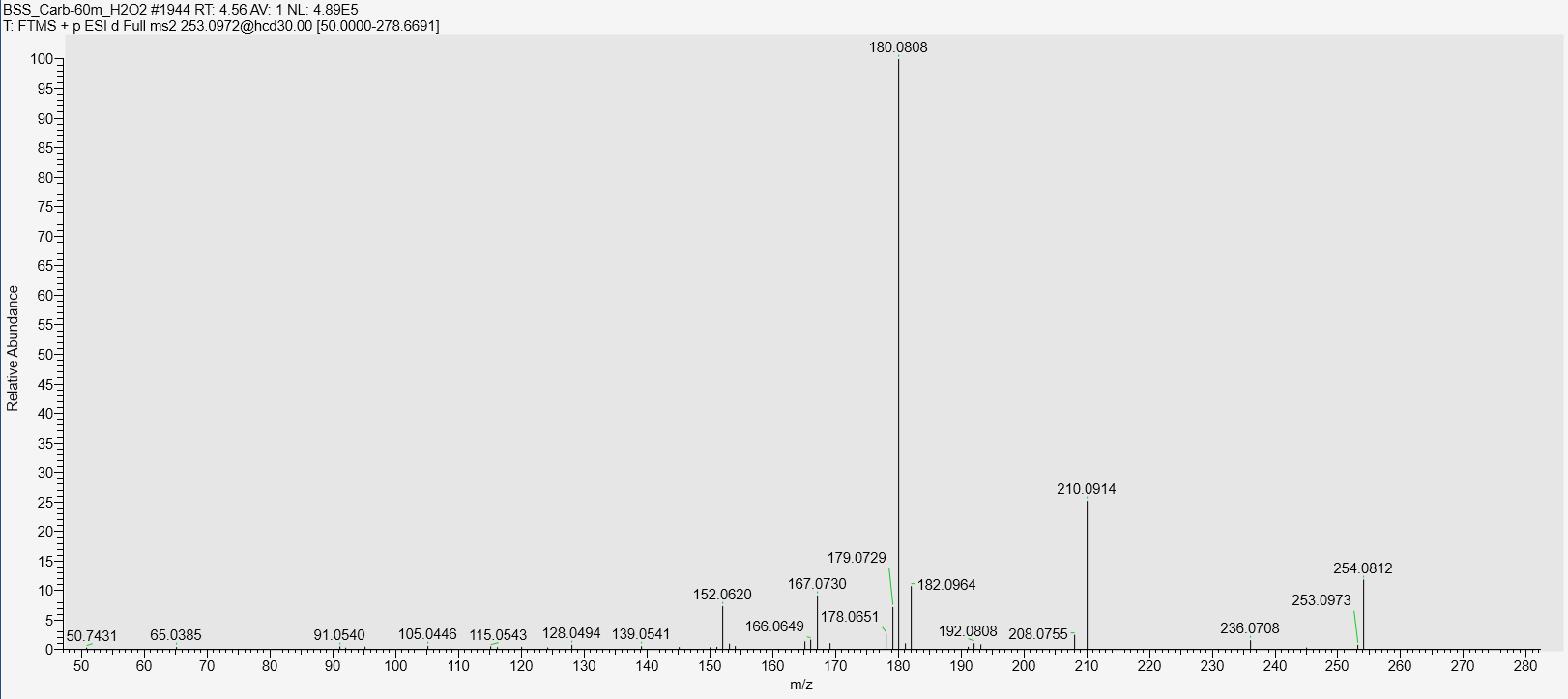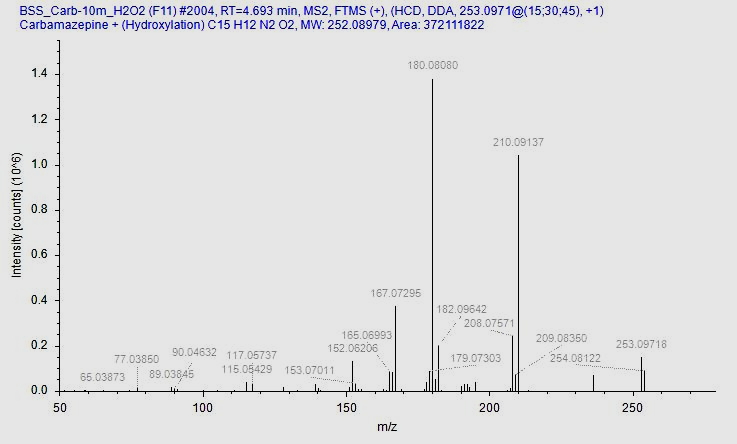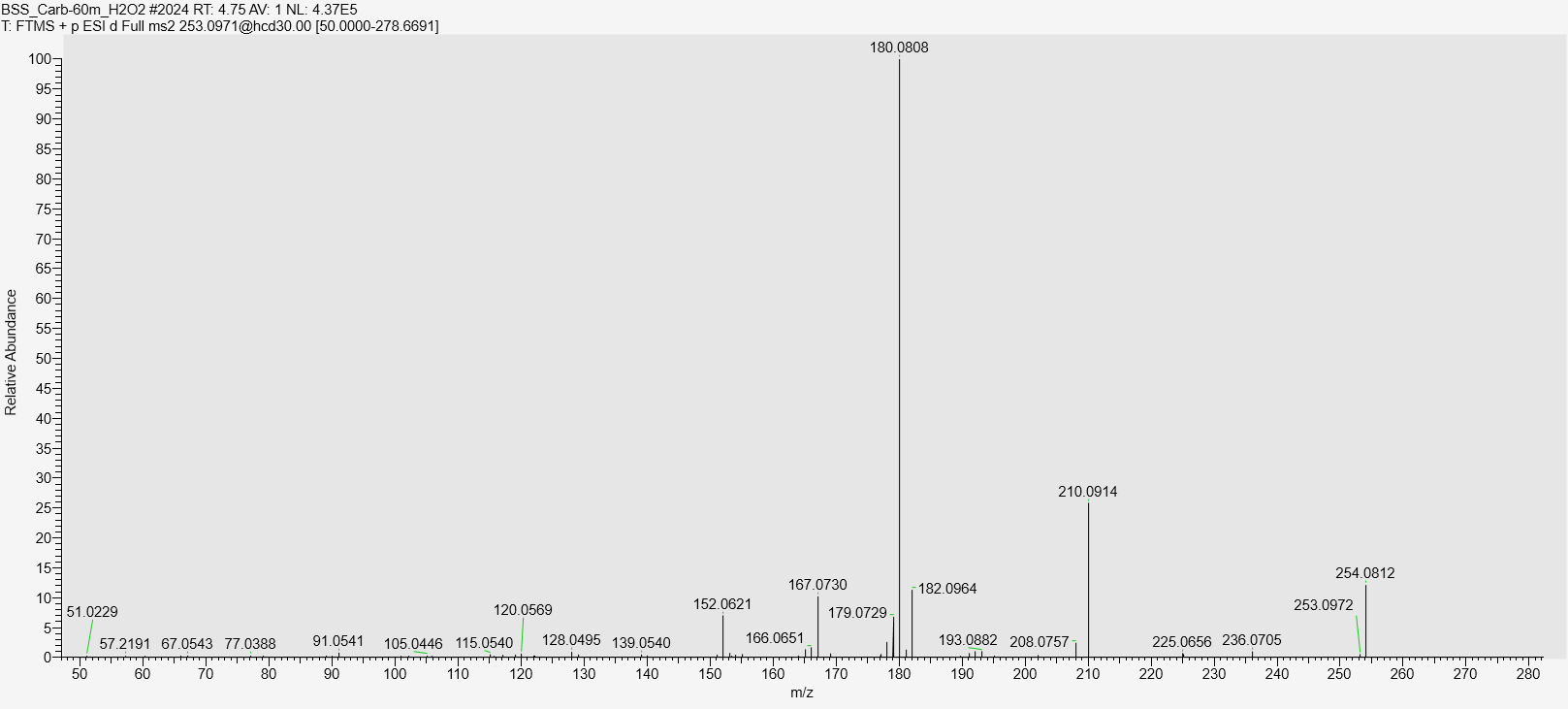 |
| 18 | C_13_H_8_ClN  *MS2 spectrum was not recorded* | [M] = 213.0345  [M+H]^+^=214.0418  [M-H] ^-^=212.0273 | 4.79 | 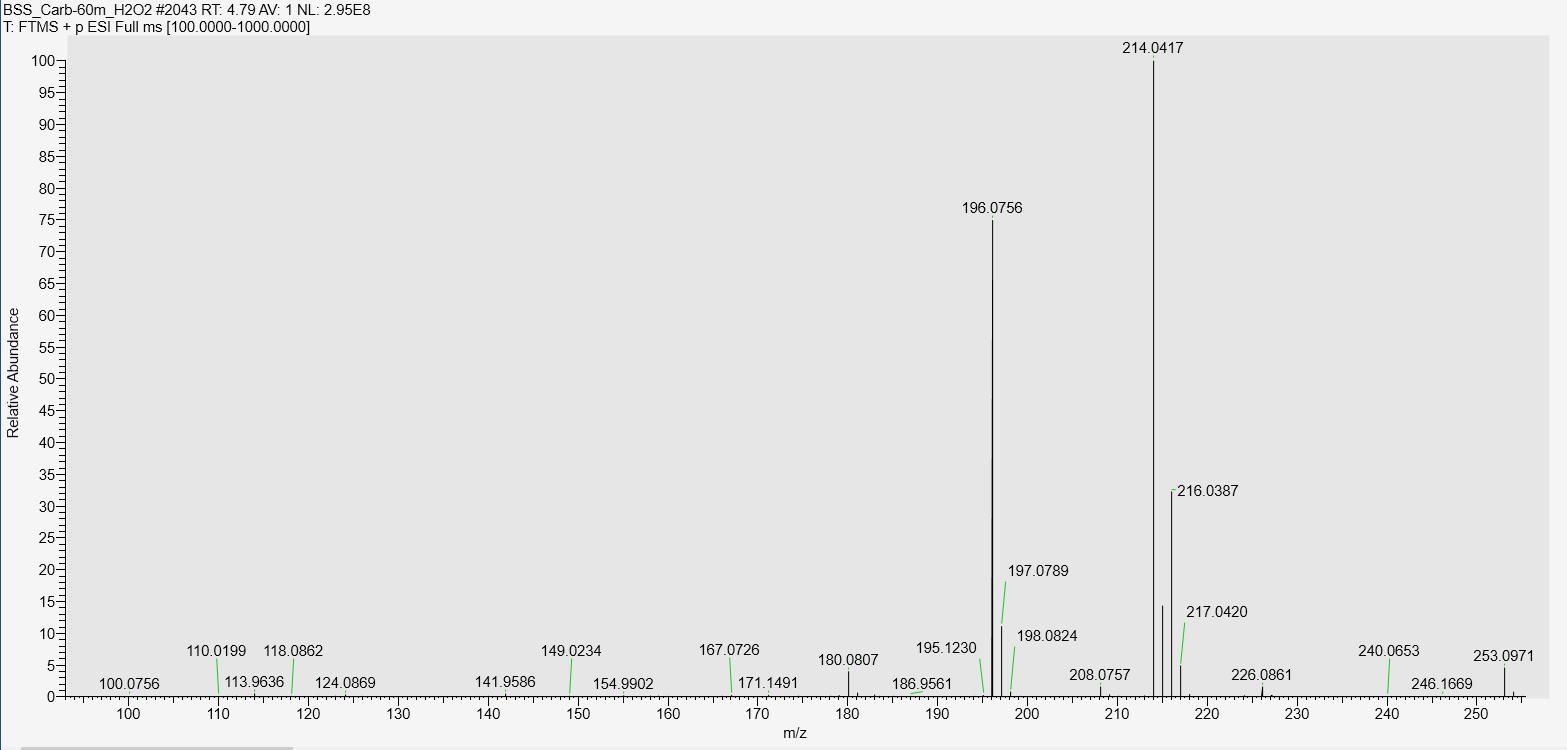 |
| 19 | C_14_H_10_ClN  10-chloro-5H-dibenzoazepine  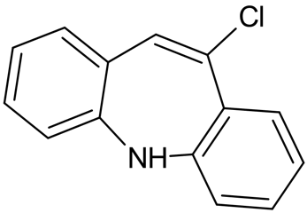 | [M] = 227.0502  [M+H]^+^ = 228.0575  [M-H] ^-^ =226.0429 | 7.5 | MS1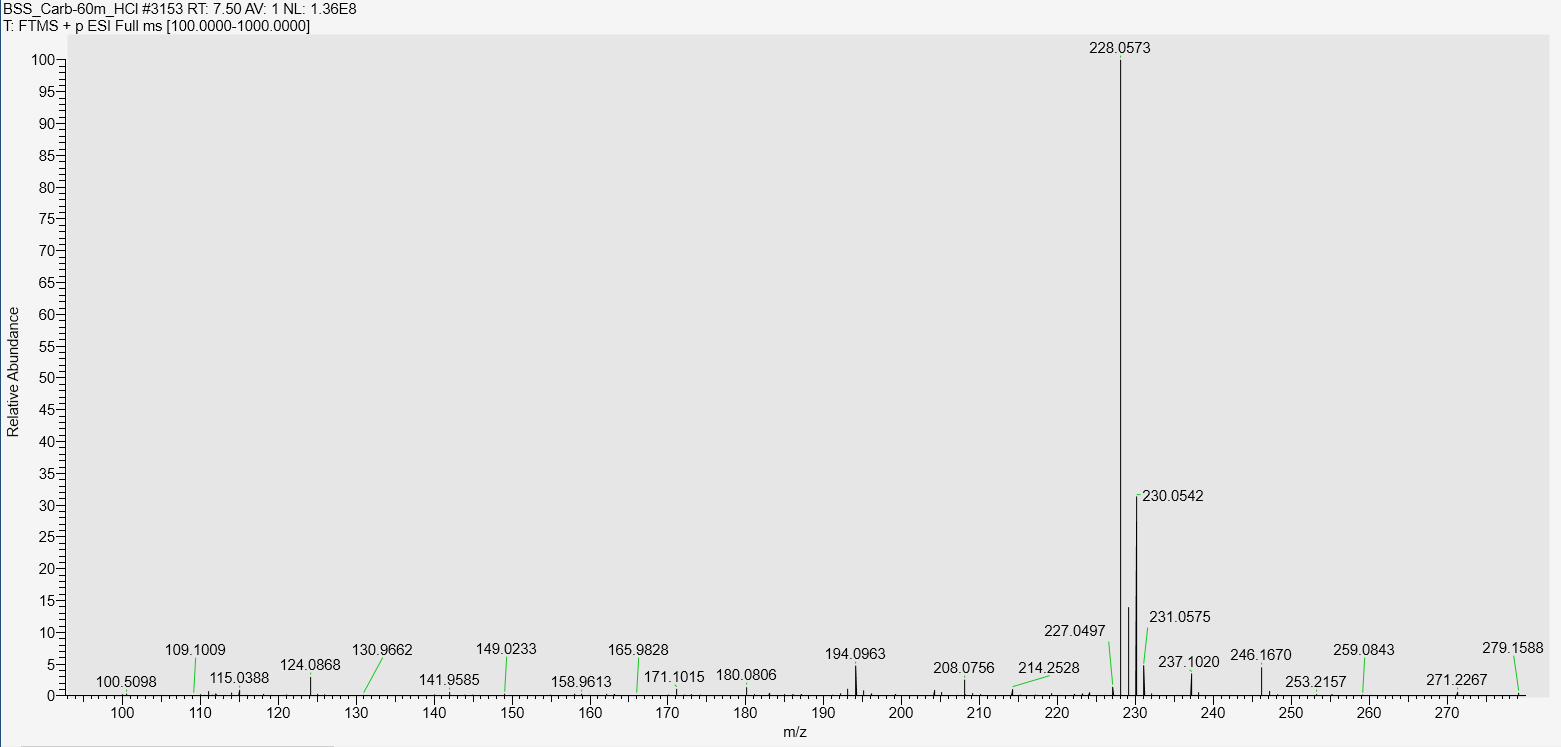  MS2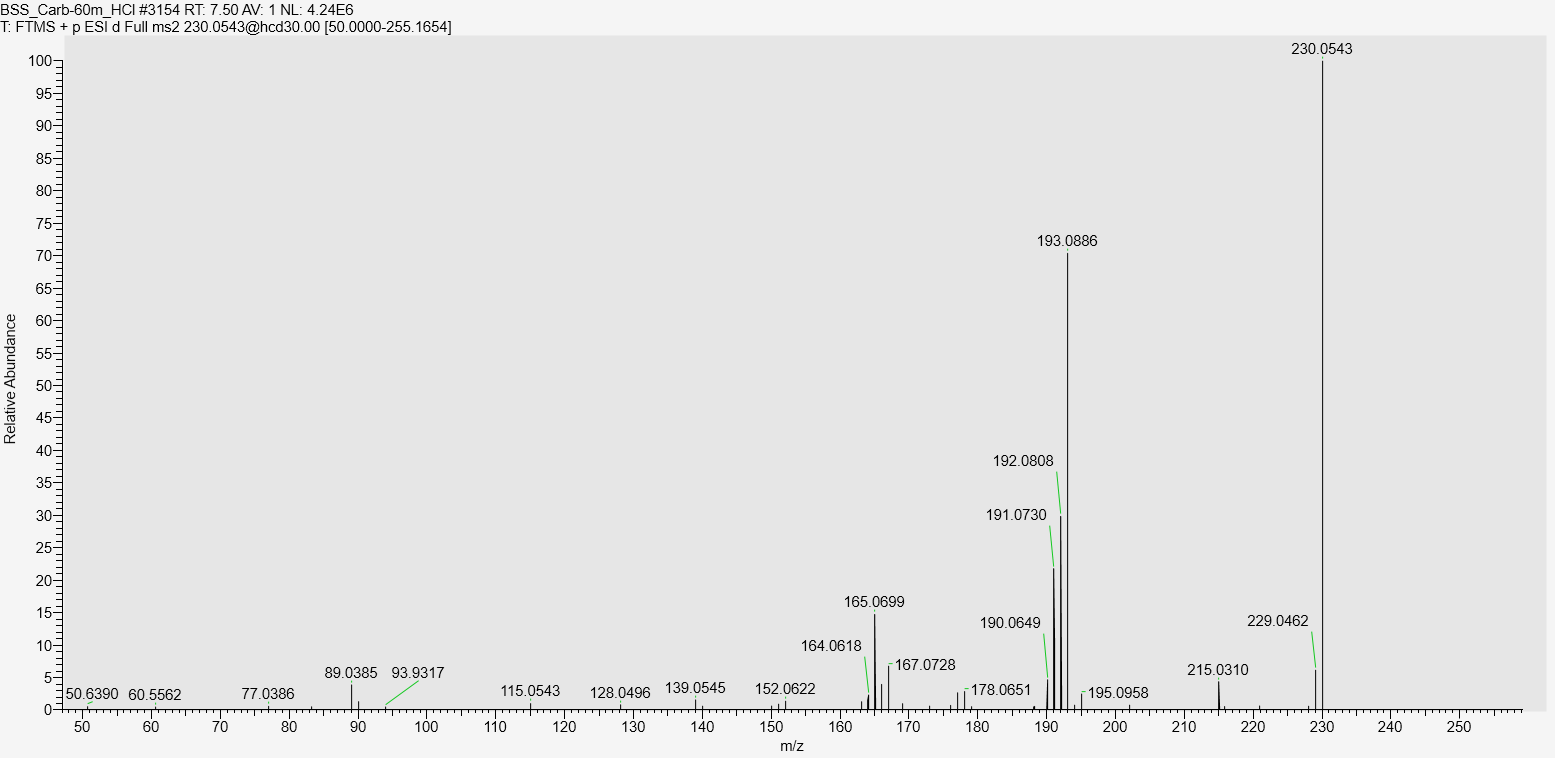 |
| 20 | C_15_H_11_ClN_2_O  10-chlorocarbamazepine  (10-Cl-CBZ)  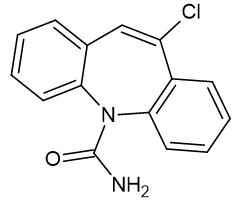 | [M] = 270.0560  [M+H] ^+^ = 271.0633  [M-H] ^-^ = 269.0487 | 5.77 | MS1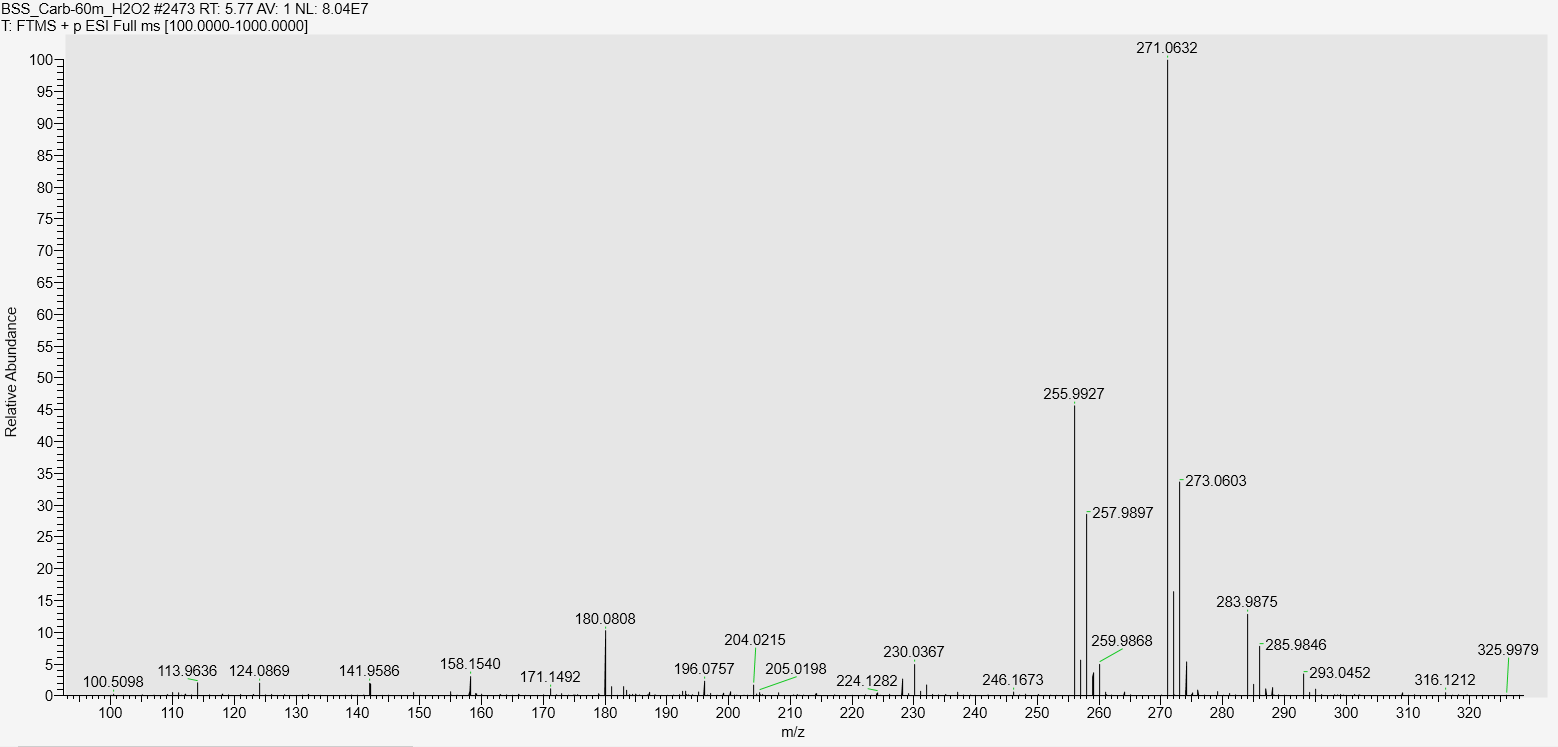  MS2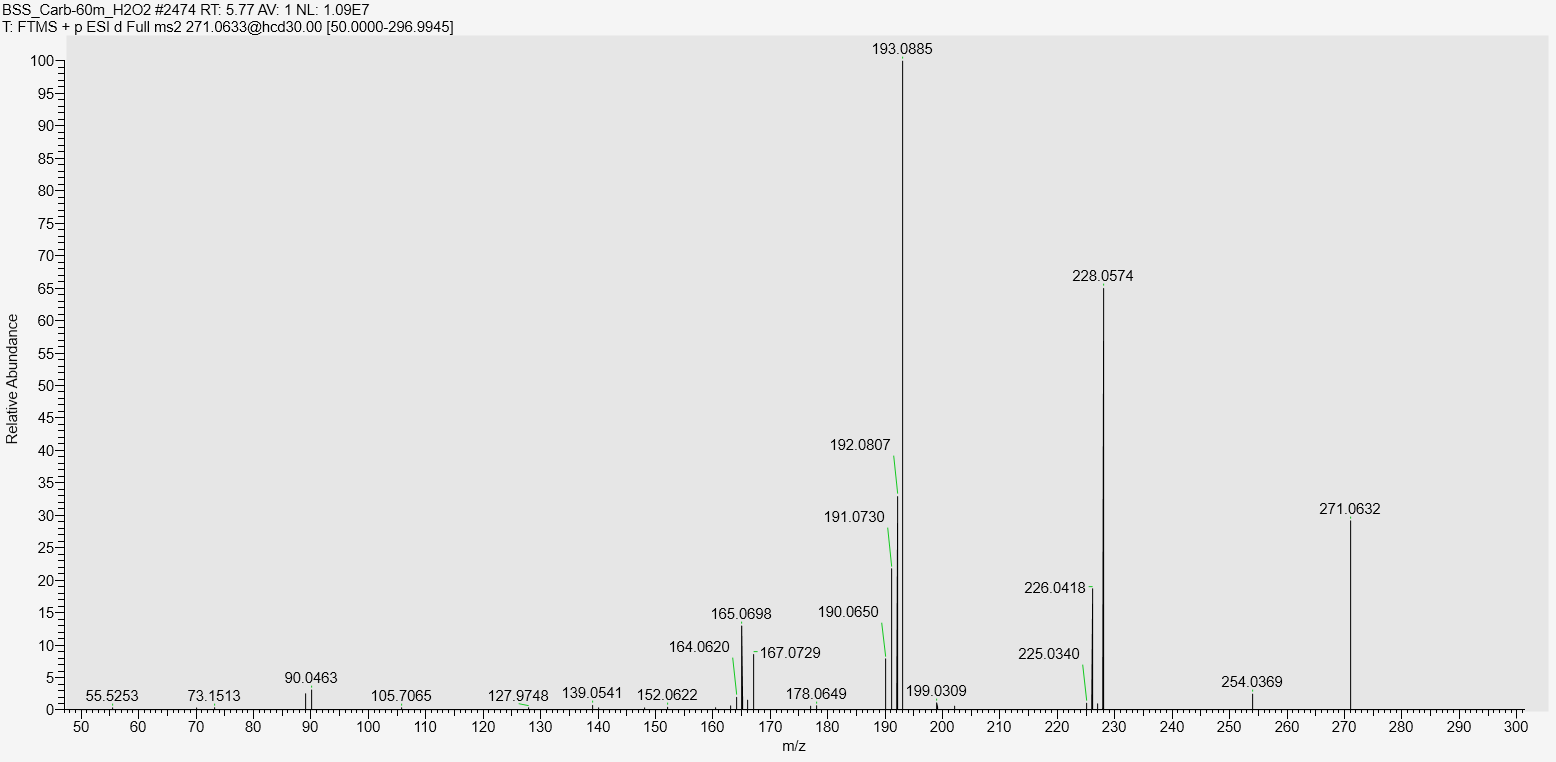 |
| 21 | C_13_H_7_Cl_2_NO  *MS2 spectrum was not recorded* | [M] = 262.9905  [M+H] ^+^ = 263.9978  [M-H] ^-^ = 261.9832 | 6.3 | 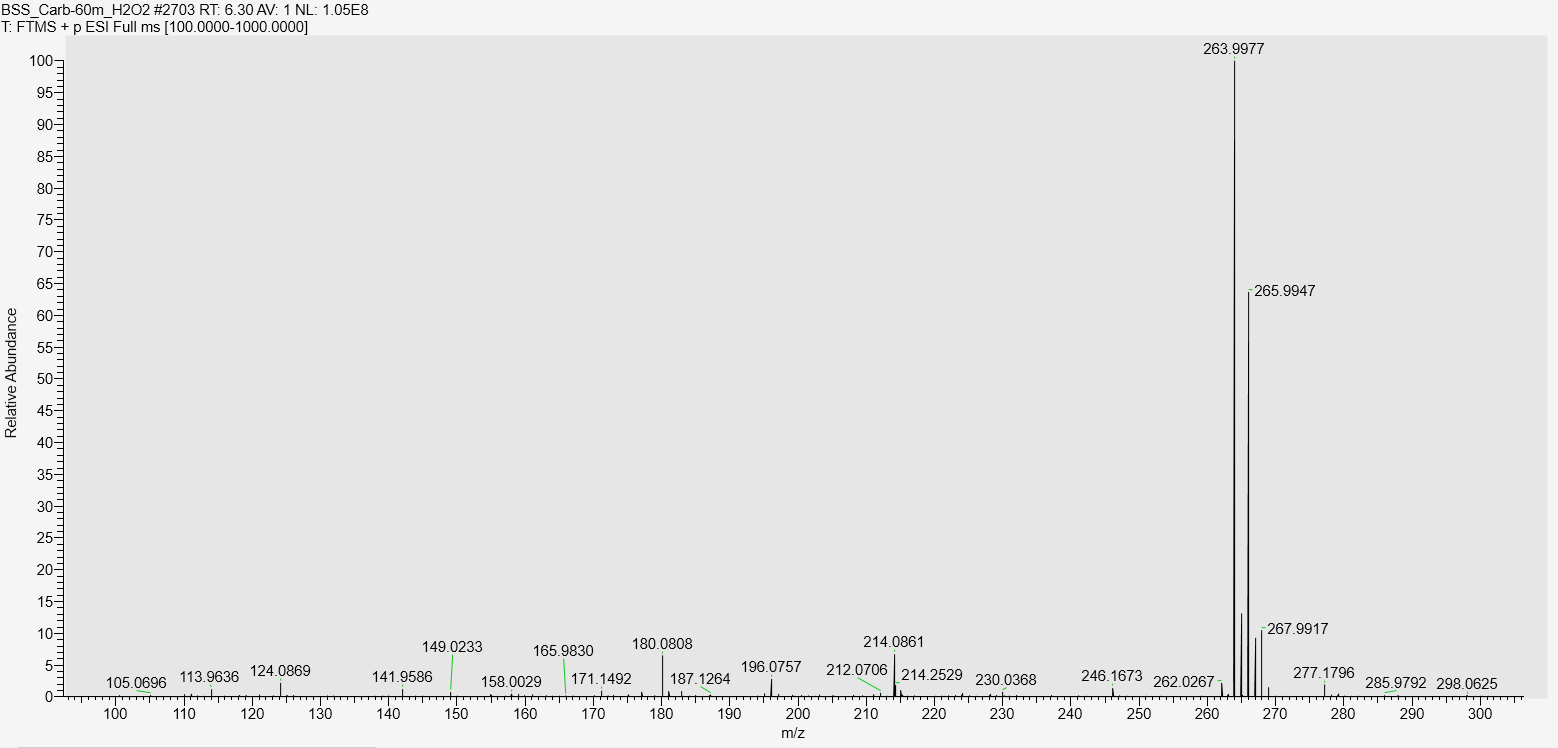 |
| 22 | C_13_H_8_ClNO  4-chloroacridin-9(10H)-one  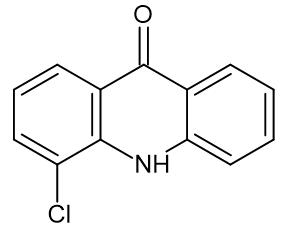 | [M] = 229.0294  [M+H] ^+^ = 230.0367  [M-H]^-^ = 228.0222 | 5.58-5.59 | MS1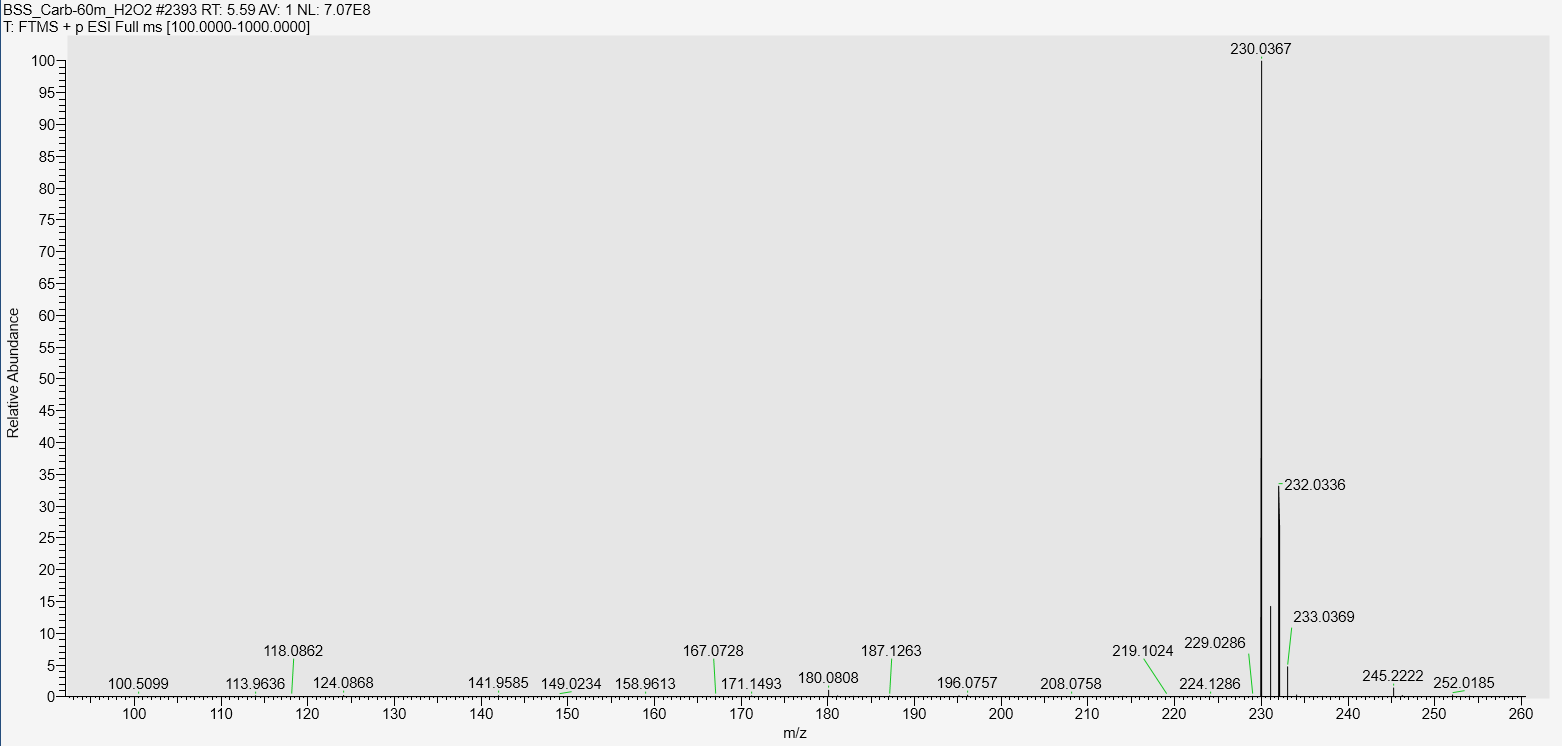  MS2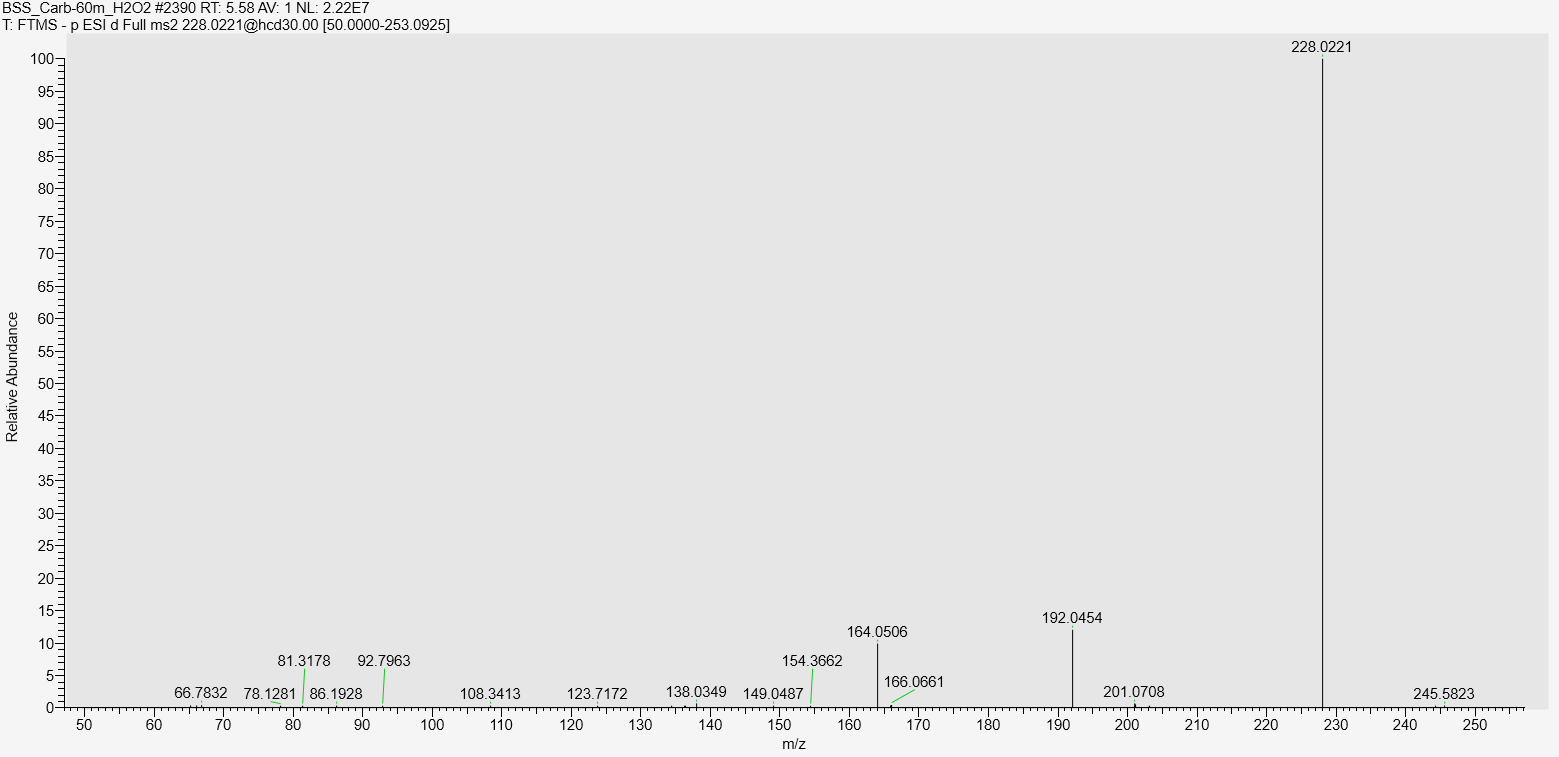 |

| А) 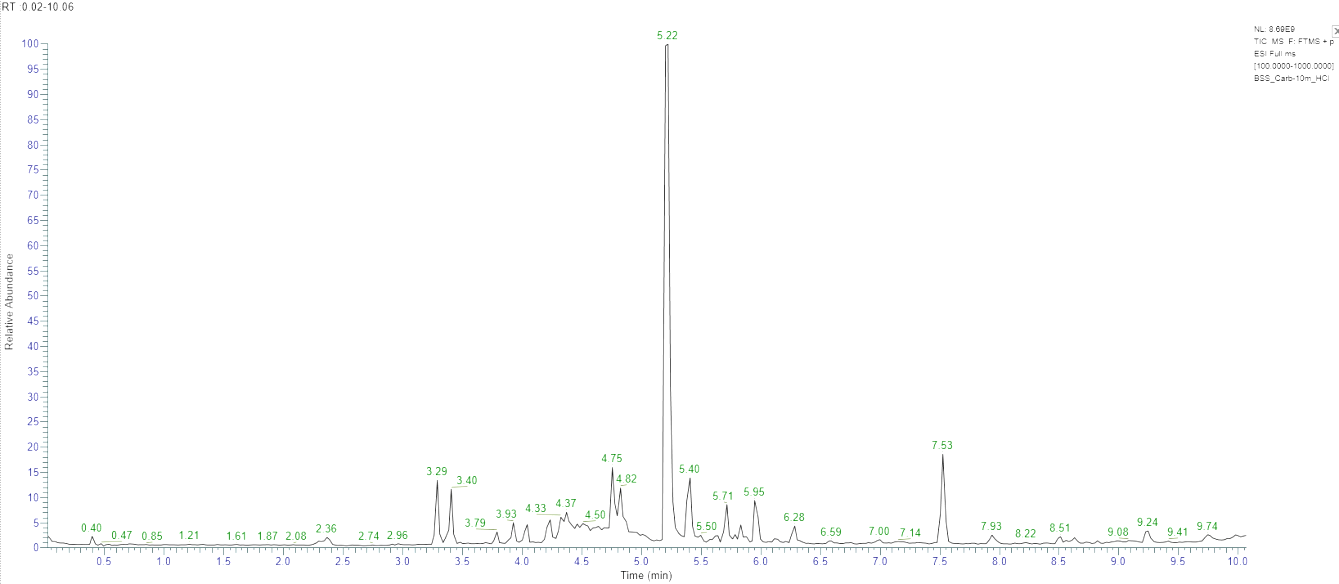 |
| --- |
| B) 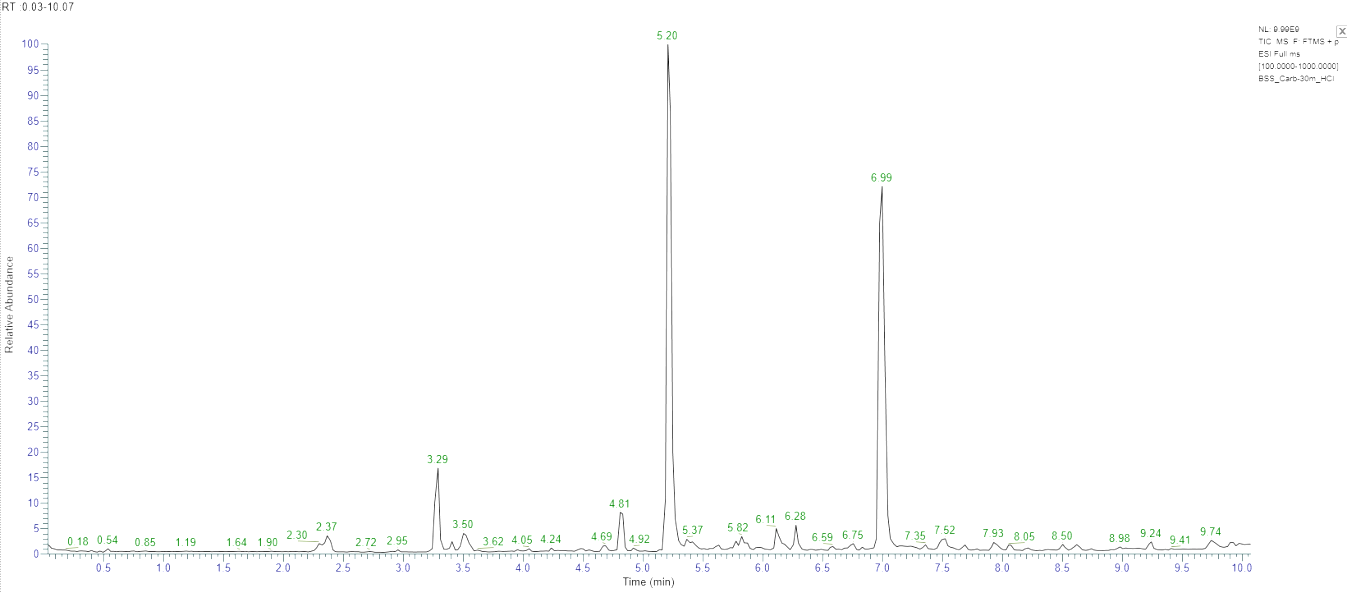 |
| C) 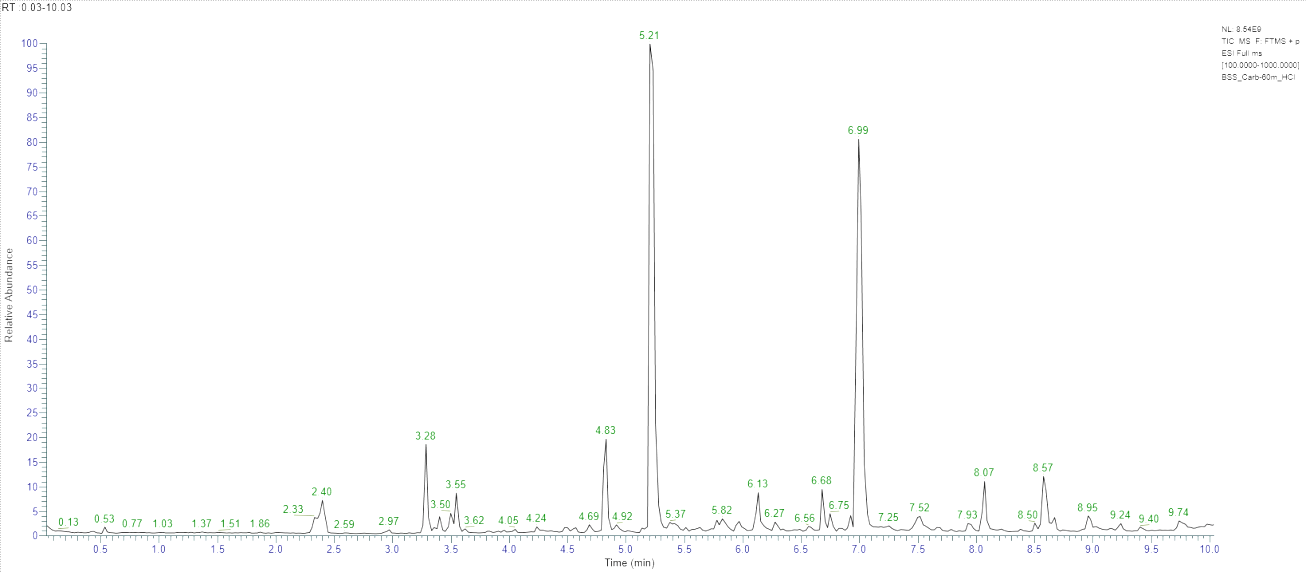 |
| Fig S1. Chromatograms of model CBZ solutions obtained after interaction with HCl for A) 10 min, B) 30 min, C) 60 min. |

| А)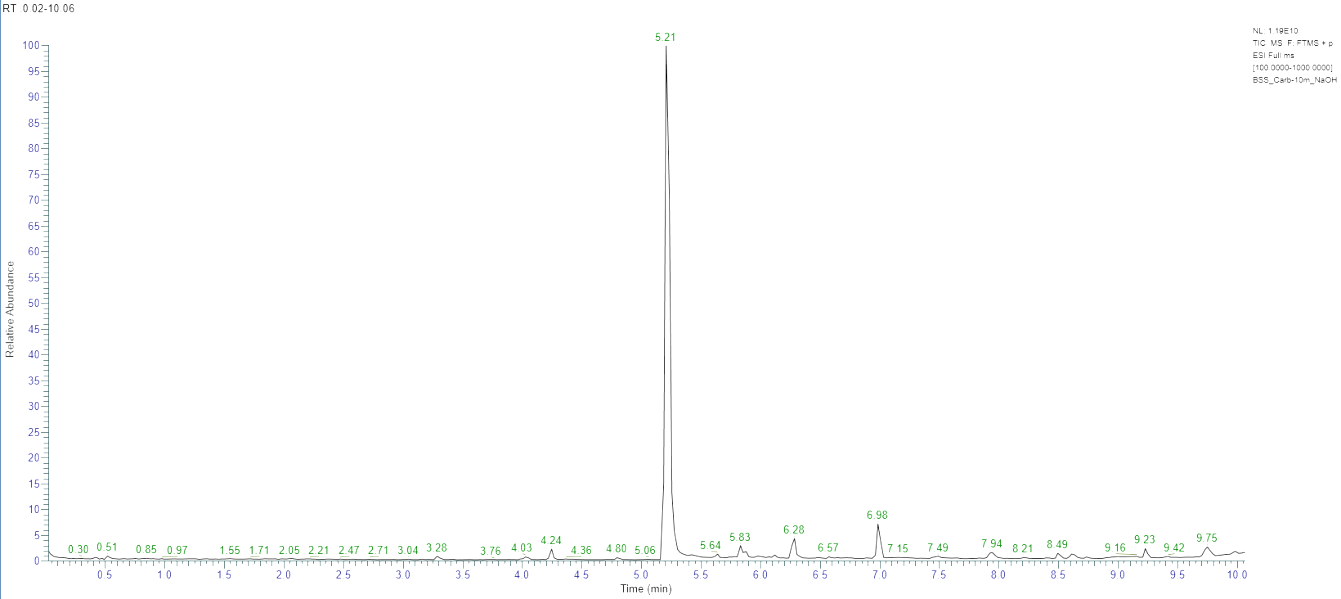 |
| --- |
| B) 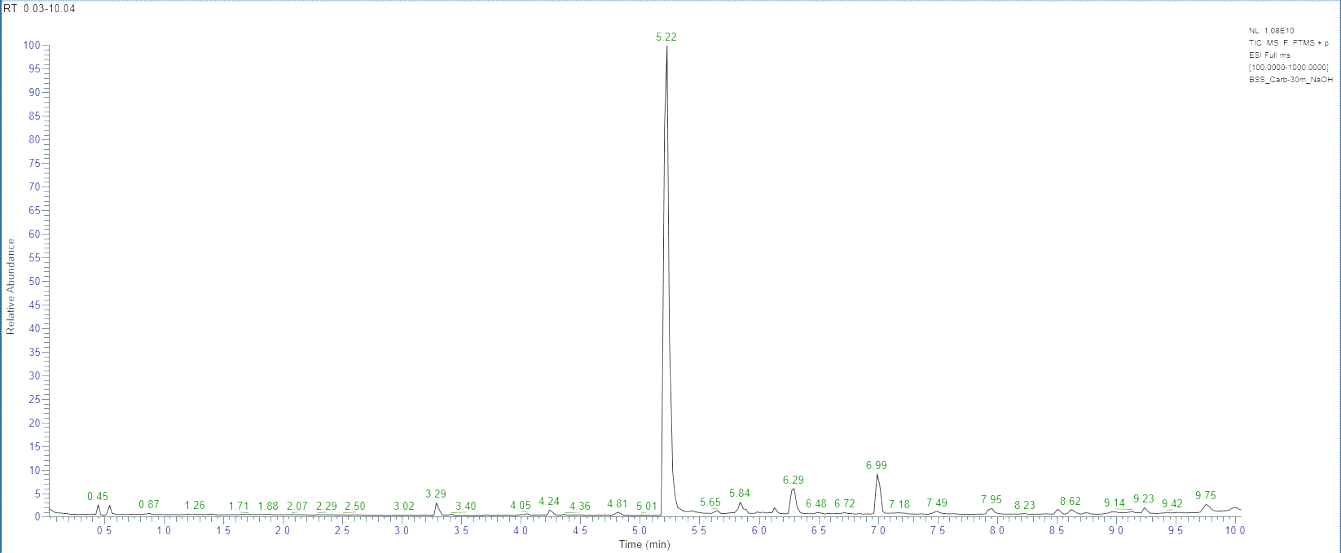 |
| C) 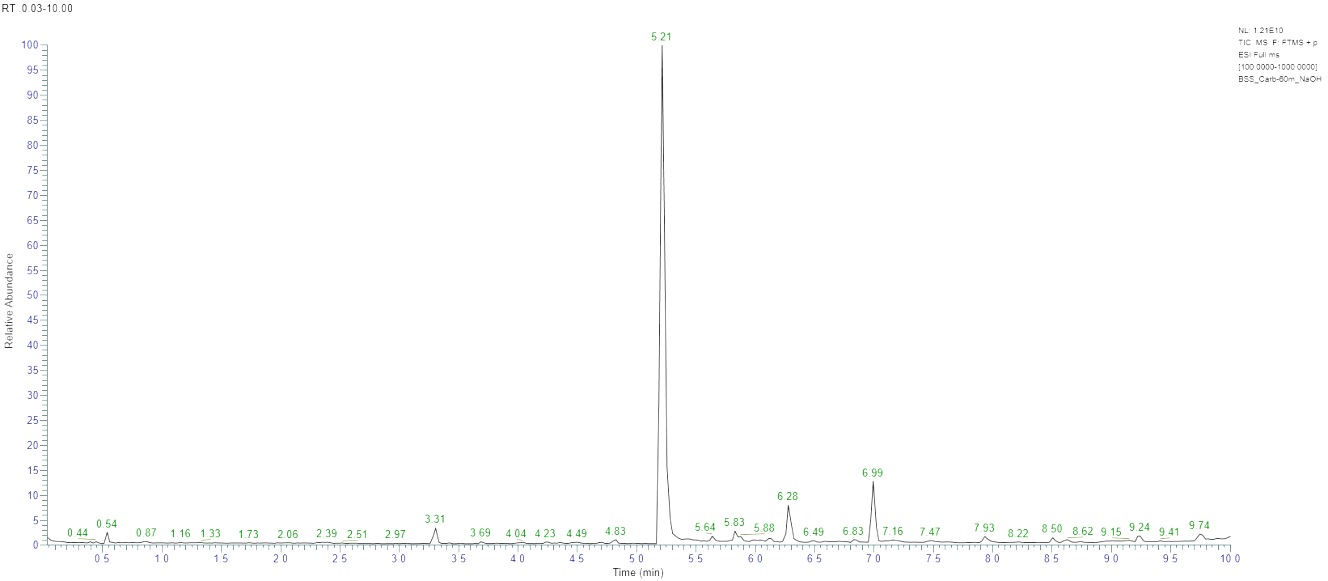 |
| Figure S2. Chromatograms of model CBZ solutions obtained after interaction with NaOH for A) 10 min, B) 30 min, C) 60 min. |

1.
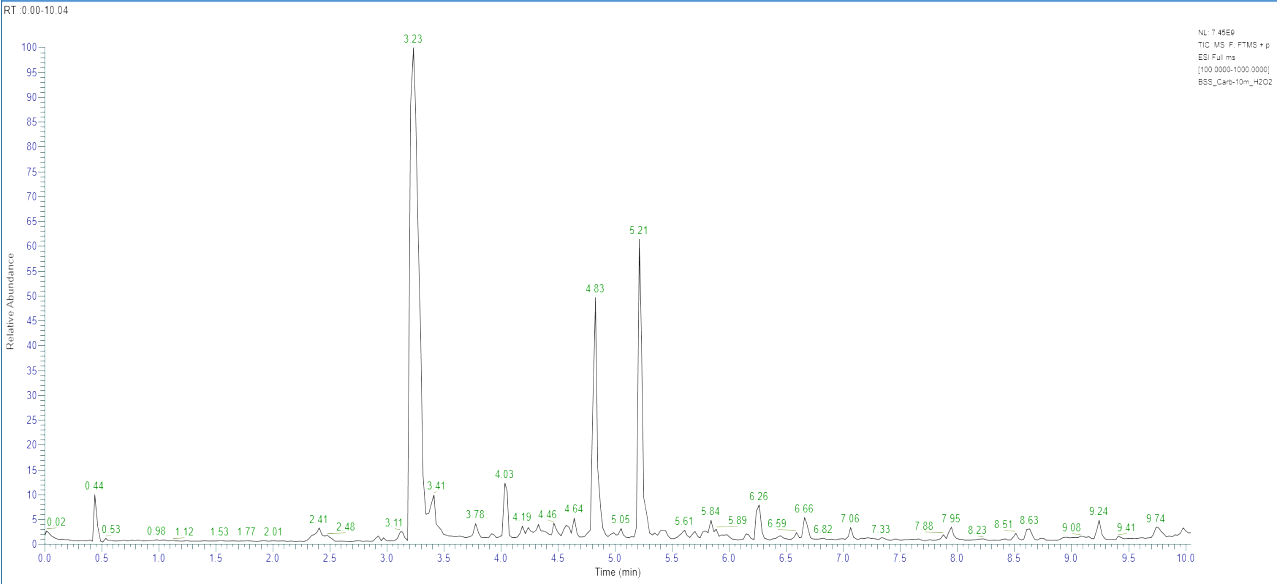

2.
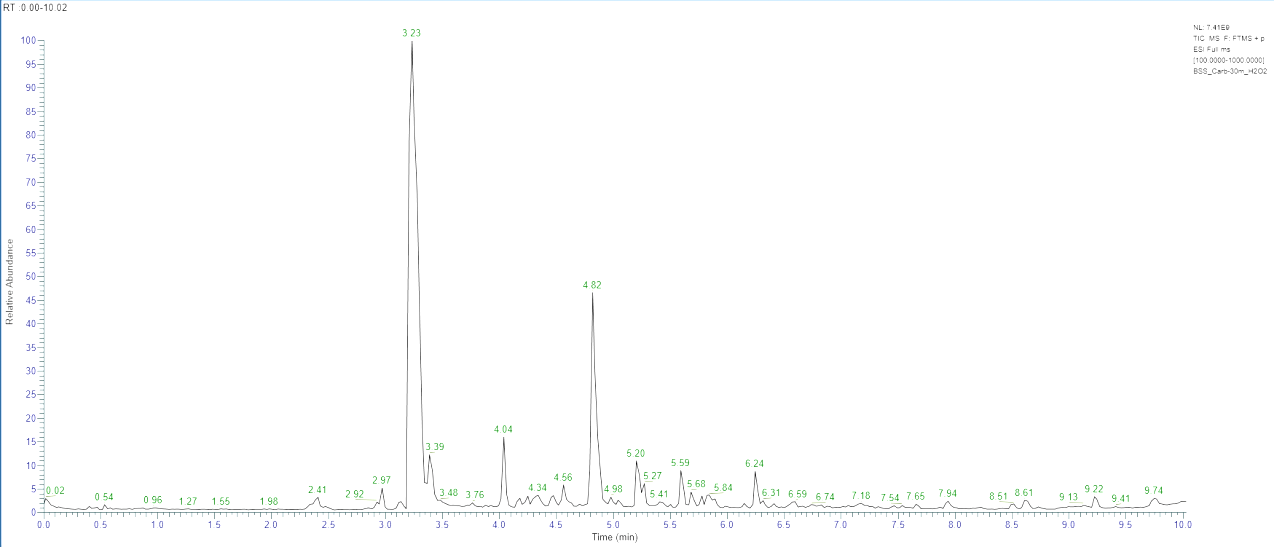

3.
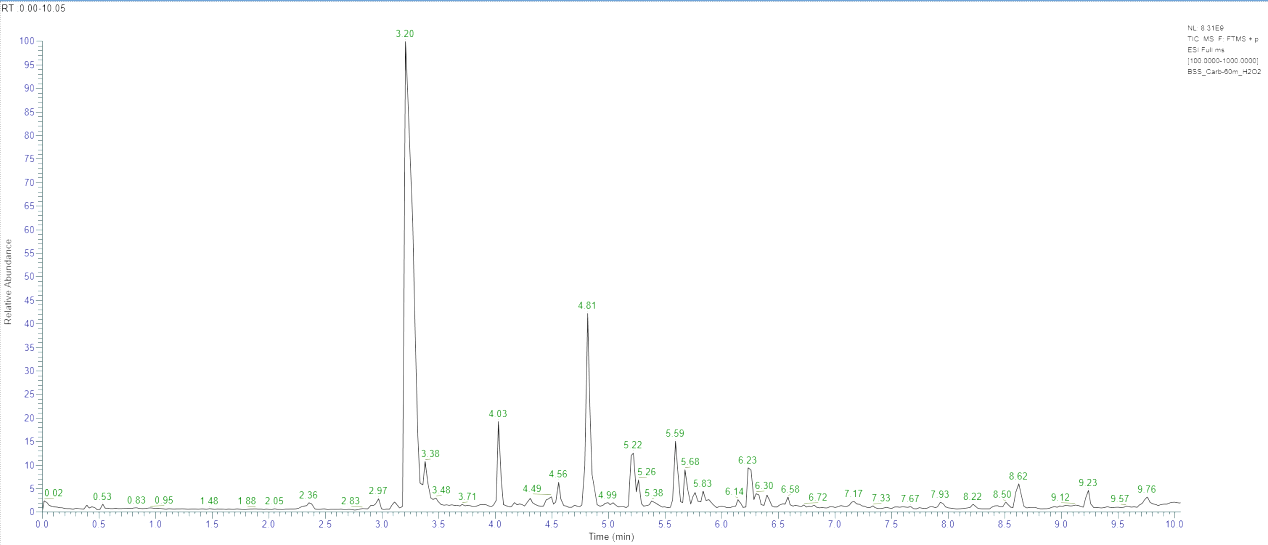


Figure S3. Chromatograms of model CBZ solutions obtained after interaction with H_2_O_2_ for A) 10 min, B) 30 min, C) 60 min.


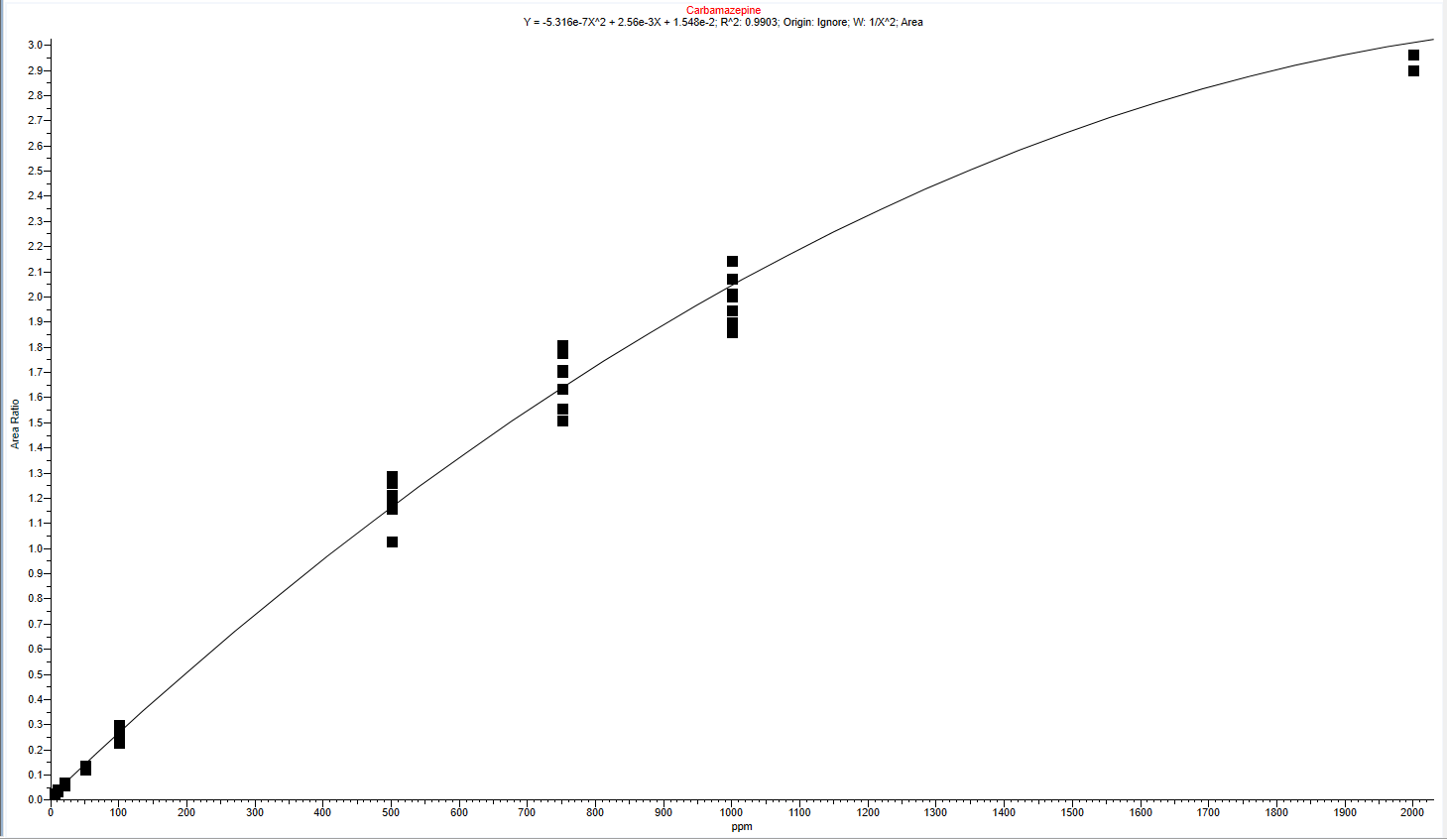


Figure S4. The calibration curve used for CBZ quantification. $Y=-5.316e^{-7}x^{2}+2.56e^{-3}x+1.548e^{-2}$ , R^2^ = 0,9903 The x-axis represents the ratio of CBZ concentration to the concentration of the internal standard, while the y-axis represents the ratio of the CBZ peak area to the internal standard peak area. The deviations of the calibration sample concentrations from their nominal values did not exceed the permissible limits (±20%) across the entire range of calibration concentrations.


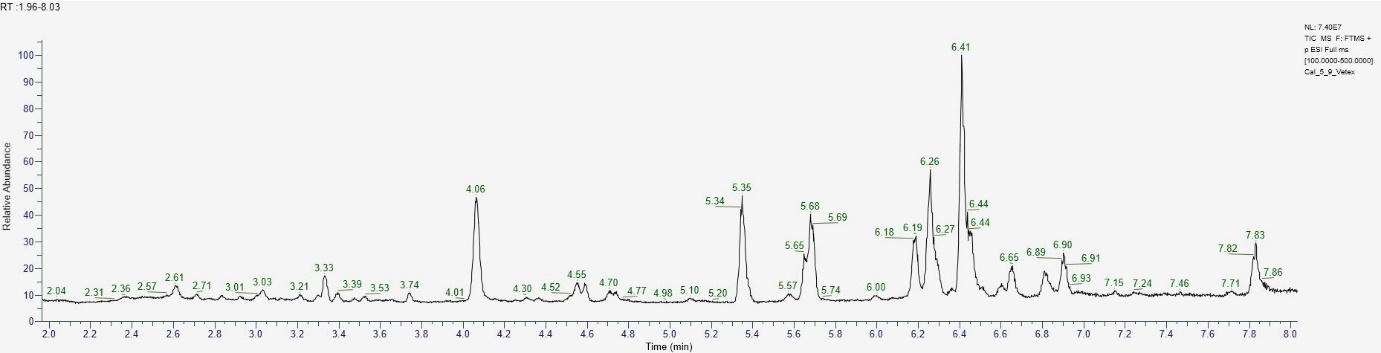


Fig. S5. Total ion current chromatogram of FSE_1.


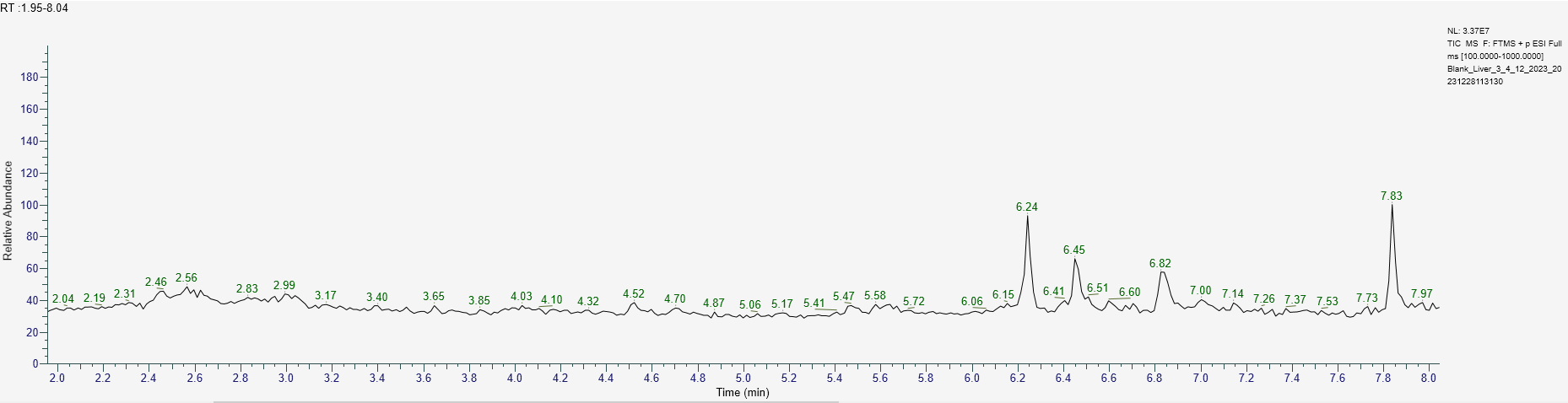


Fig. S6. Total ion current chromatogram of blank liver sample.

Table S4. **Signal parameters at the LOD level (1 ng/g) during the fifth analytical cycle.**

| **Parameter** | **Value** |
| --- | --- |
| Measured concentration, ng/g | 1.04 |
| Retention time, min | 5.35 |
| Peak area | 215,340 |
| Peak height | 112,193 |


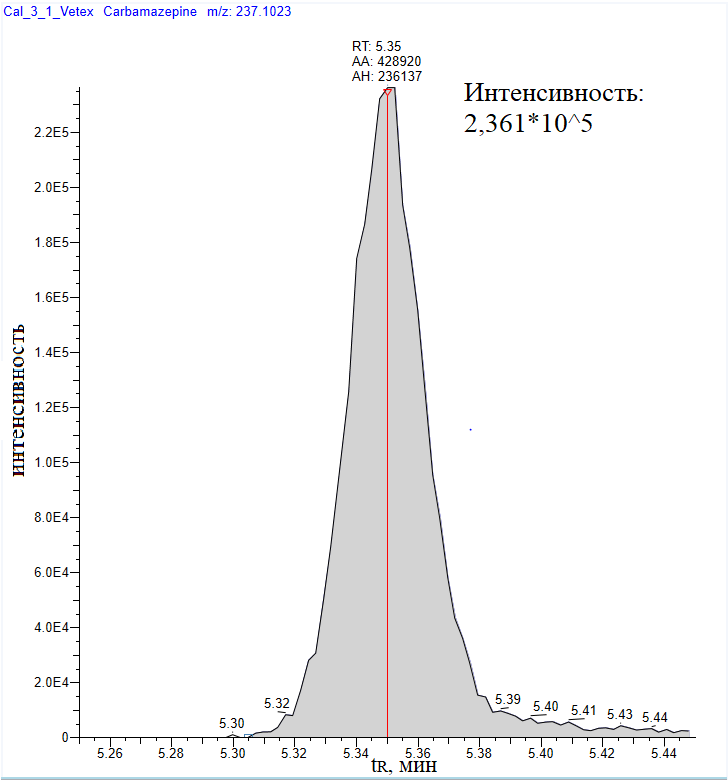

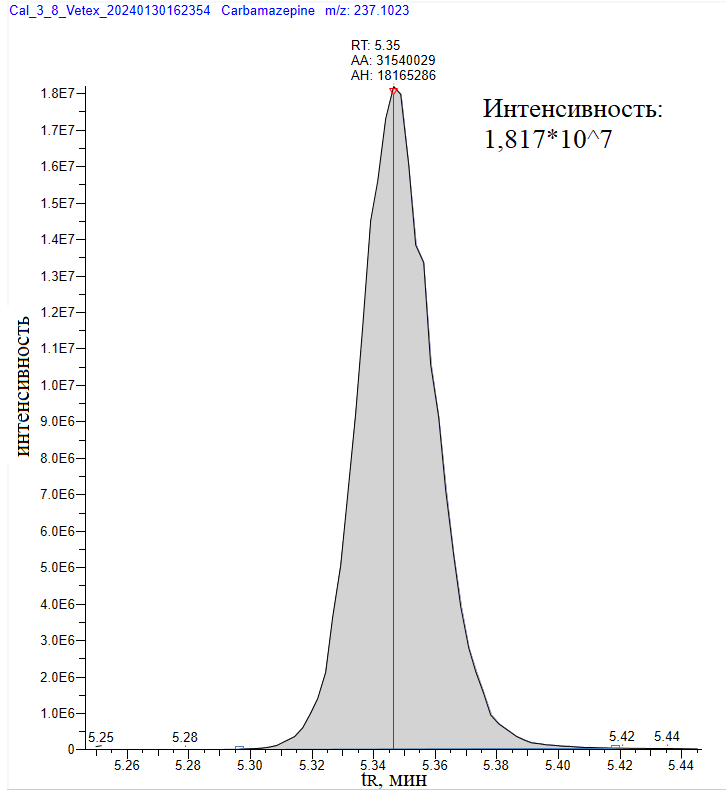


B

Intensity – 1.817E7

A

Intensity – 2.361E5

**Fig. S7.** A fragment of the chromatogram of a sample containing CBZ at the LOQ level (5 ng/g) **(A)** is compared with a fragment of the chromatogram of calibration sample No. 8 containing 1000 ng/g of CBZ **(B)**
